# A pooled analysis of the association between sarcopenia and osteoporosis

**DOI:** 10.1097/MD.0000000000031692

**Published:** 2022-11-18

**Authors:** Xiaochao Yu, Shuo Sun, Shaoxiong Zhang, Qinggang Hao, Boheng Zhu, Yirong Teng, Qing Long, Shujun Li, Yan Lv, Qiaoning Yue, Sheng Lu, Zhaowei Teng

**Affiliations:** a Kunming Medical University, Kunming, Yunnan, China; b The First People’s Hospital of Yunnan Province, Kunming, Yunnan, China; c School of Life Sciences, Yunnan University, Kunming, Yunnan, China; d The Second Affiliated Hospital of Kunming Medical University, Kunming, Yunnan, China.

**Keywords:** Meta-analysis, Osteoporosis, Risk, Sarcopenia

## Abstract

Sarcopenia is a progressive generalized skeletal muscle disorder that causes the accelerated loss of muscle mass and function. Osteoporosis is a systemic condition of the skeleton that results in low bone mass and quality. Several studies have suggested that osteoporosis and sarcopenia are interrelated; however, a few studies indicate the lack of a significant association between sarcopenia and osteoporosis. We aimed to evaluate the association between sarcopenia and osteoporosis via a systematic review and pooled analysis.

**Methods:**

From the inception of the PubMed and Embase databases until September 2022, we conducted a systematic search for studies evaluating the relationship between sarcopenia and osteoporosis. Study appraisal and synthesis methods: We included observational studies that provided 95% confidence intervals (CIs) and risk estimates. Two reviewers independently extracted data and assessed the quality of the research. The random-effects model was applied to the pool analysis, and the odds ratios (ORs) and 95% CIs were finally calculated.

**Results:**

The primary statistic was the mutual risk between sarcopenia and osteoporosis. According to the inclusion criteria, 56 studies (796,914 participants) were finally included. Sarcopenia was significantly correlative to the risk of osteoporosis (OR, 3.06; 95% CI, 2.30–4.08), and each standard deviation increase in relative appendicular skeletal muscle mass was significantly related to a decreased risk of osteoporosis (OR, 0.65; 95% CI, 0.56–0.75). Osteoporosis observably referred to a higher risk of sarcopenia (OR, 2.63; 95% CI, 1.98–3.49).

**Conclusion:**

Our research indicated that sarcopenia and osteoporosis are highly positively correlated. Osteoporosis is closely associated with the risk of sarcopenia. Our finding highlights the importance of sarcopenia screening for those at risk of osteoporosis, and vice versa. However, heterogeneity was noted among the studies, and this might have influenced the accuracy of the results. Therefore, the results of our study should be interpreted with caution.

## 1. Introduction

Sarcopenia is a skeletal muscle disease that involves the progressive loss of muscle mass and function throughout the body.^[1]^ Osteoporosis is a systemic condition of the skeleton that results in low bone mass and quality.^[2]^ Sarcopenia and osteoporosis have evolved into worldwide health issues, are both associated with aging, impaired quality of life, and adverse health situations, and they regularly occur simultaneously.^[1–4]^

Muscle and bone, which can influence each other, can both be viewed from the perspectives of biomechanics and biochemistry.^[5]^ Several studies have investigated the relationship between sarcopenia and osteoporosis and have suggested that osteoporosis can increase the risk of sarcopenia.^[6–8]^ Other studies have revealed that individuals with sarcopenia present with a significantly elevated risk of osteoporosis.^[9–11]^ A study of community-dwelling older women reported that the loss of skeletal muscle mass was an independent risk factor for osteoporosis.^[12]^ However, a few studies reported the lack of a significant association between sarcopenia and osteoporosis.^[7,13,14]^ Thus, a pooled analysis was performed to reveal the interrelationship between sarcopenia and osteoporosis in order to guide clinical practice.

## 2. Material and methods

This pooled analysis as performed based on the Meta-analysis of Observational Studies in Epidemiology (MOOSE) guidelines and the Preferred Reporting Items for Systematic Reviews and Meta-Analyses (PRIMSA) (File S1, Supplemental Digital Content, http://links.lww.com/MD/H885).^[15,16]^ We registered the systematic review protocol in PROSPERO on October 1, 2020 (CRD420167604). Ethics Approval/ Institutional Review Board (IRB) was not required for this study.

### 2.1. Search strategy and selection of eligible studies

PubMed and Embase were searched from their inception to September 1, 2022, with no language restrictions. We focused on the correlation between sarcopenia and osteoporosis in humans. Our core search consisted of the following keywords: “sarcopenia” and “osteoporosis” (File S2, Supplemental Digital Content, http://links.lww.com/MD/H886). We included all observational studies such as cohort studies, cross-sectional studies, case-control studies, etc. Two authors (XCY and QGH) independently reviewed the titles and abstracts of all studies identified through the database and independently selected relevant studies according to the inclusion criteria. If a dataset was repeatedly analyzed in studies, we chose studies that were more recent or had the largest sample sizes and data considerations. We found additional pertinent studies by reviewing the reference lists of identified studies. For cases with missing necessary data for analysis, we attempted to contact the author by e-mail. We included studies that either directly reported risk estimates (relative risks, odds ratios [ORs], or hazard ratios) with 95% confidence intervals (CIs) or provided sufficient data to calculate these estimates. We excluded studies that were published without adequate data and for which data could not be obtained from the authors. Based on the Strengthening the Reporting of Observational Studies in Epidemiology statement, we estimated the quality of the included literature.^[16]^ Disagreements in the selection process were resolved by the corresponding authors (i.e., TZW and LS) until an agreement was reached.

### 2.2. Data extraction

Two teams extracted all the data. If 1 study included more than 1 cohort, we pooled the studies and considered each cohort an independent study. Any disagreement was settled by consensus or by the corresponding authors. We extracted study parameters such as the name of the first author, year of publication, population/region in which the study was performed, study design, sample size, participant sex and age, outcome measurements related to risk estimates with 95% CIs, adjustment factors, and interaction risk between sarcopenia and osteoporosis.

### 2.3. Data analysis

A pooled analysis was performed to investigate the association between sarcopenia and osteoporosis. The pooled OR and 95% CI were estimated from the adjusted ORs and 95% CIs reported in the studies or from the ORs and 95% CIs calculated by the chi-squared (*χ*^2^) test. We applied Cochran *Q* and *I*^2^ statistics to compute statistical heterogeneity^[17]^; 25%, 50%, and 75% were considered cutoff points for low, medium, and high heterogeneity of data, respectively. When the *P*-value was < .1 and the I^2^ value was > 50%, the data were considered heterogeneous, and a random-effects model^[18]^ was applied. In addition, we performed subgroup analyses by study design, study region, sex, and sarcopenia definitions to identify the source(s) of heterogeneity. Studies used different definitions of sarcopenia including that of the Asia Working Group for Sarcopenia (AWGS),^[19]^ European Working Group on Sarcopenia in Older persons (EWGSOP),^[20]^ and others.^[13,21]^ Sensitivity analysis was applied (by excluding each study in turn) to estimate the influence of each individual study on the pooled result in order to determine the stability of the results. The subgroup analysis was restricted to sex, study design, region, and diagnostic criteria of sarcopenia. Begg test was used to assess the potential for publication bias,^[22]^ and if publication bias was noted, the trim-and-fill method^[23]^ was used to assess the influence of the bias on the stability of the results. STATA version 12.0 (College Station, TX) was used to analyze the data.

## 3. Results

### 3.1. Selected studies

**Figure 1.**
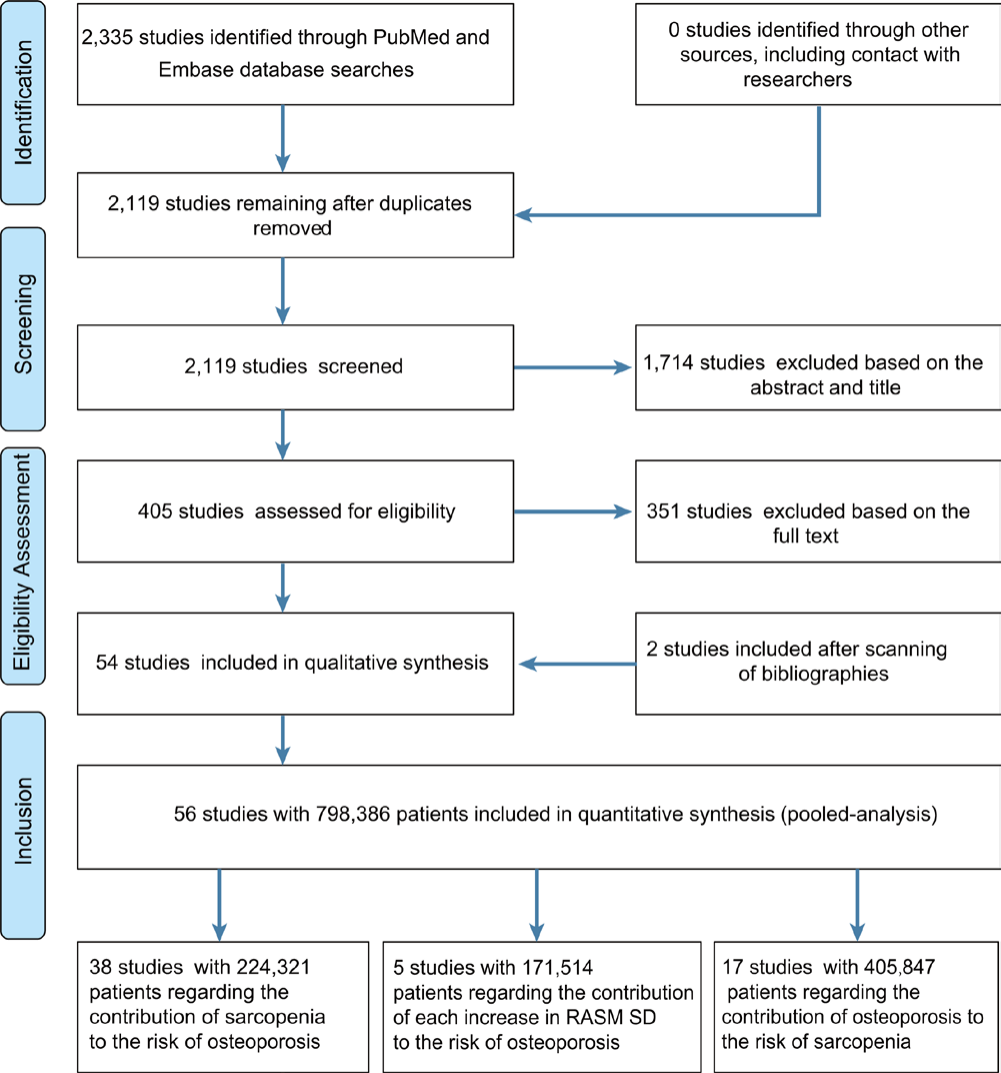
PRISMA diagram of study selection.

The PubMed and Embase databases (from their inception to September 1, 2022) were searched for observational studies on the relationship between sarcopenia and osteoporosis. Initially, 2335 studies were obtained, and after removing 216 duplicates, 2119 were identified. After screening the titles and abstract, 1714 studies were excluded; 405 needed reading of the entire article. After the data had been screened, 54 studies fulfilled the established criteria. The bibliographies of relevant studies were also searched, and 2 additional studies^[24,25]^ were identified to be eligible. Ultimately, 56 studies^[6–14,21,24–69]^ involving 796,914 participants were included. Of these, 38 studies^[6,9–11,13,21,25,27–29,31–33,35,37–39,41–43,45,47–49,51,52,54–56,58,59,61–63,65–68]^ comprising 50 independent studies examined the contribution of sarcopenia to osteoporosis, 17 studies^[6–8,12,14,24,26,30,34,36,44,50,52,53,57,64,69]^ with 405,847 participants examined the contribution of osteoporosis to sarcopenia, and 5 studies^[61–65]^ comprising 7 independent studies with 171,514 participants examined the decrease in osteoporosis risk for each standard deviation (SD) increase in relative appendicular skeletal muscle mass (RASM) (Fig. 1). Tables S1–S3, Supplemental Digital Content, http://links.lww.com/MD/H887 outline the study characteristics and quality, respectively.

### 3.2. Outcomes regarding the association of sarcopenia to the risk of osteoporosis

**Figure 2.**
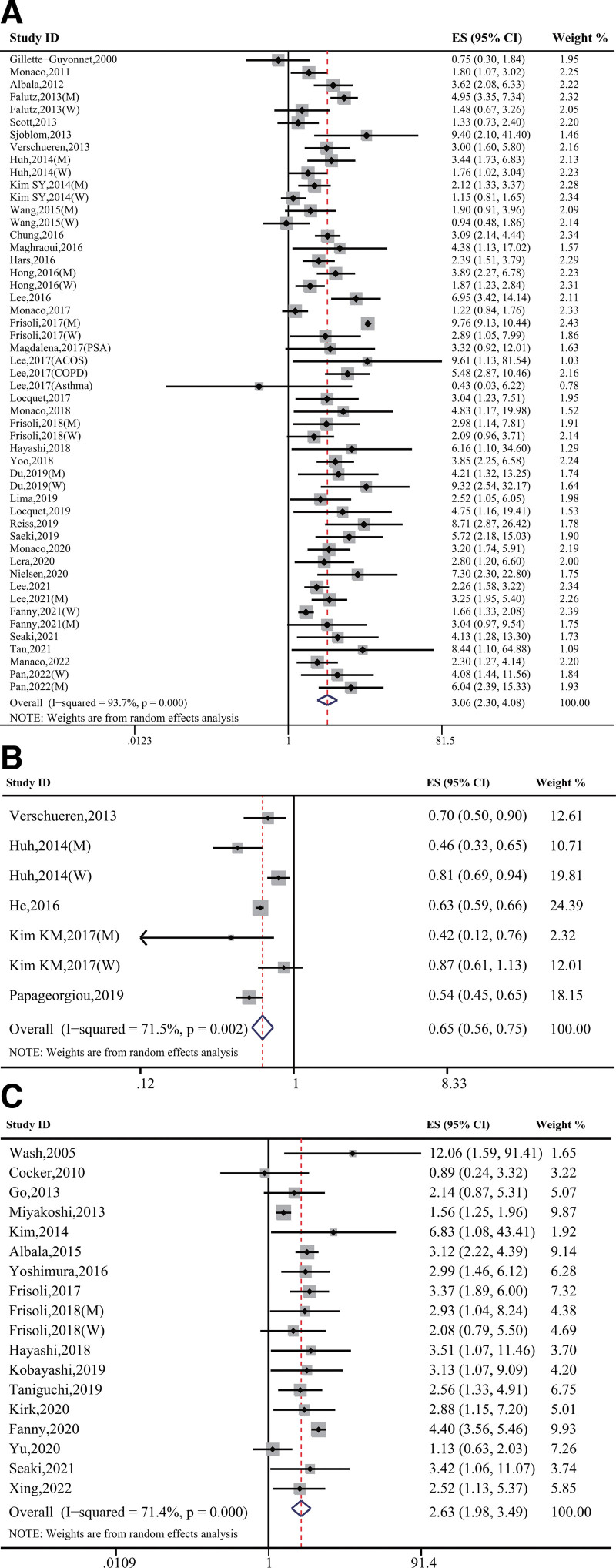
Forest plots of the estimated effects. (A) Forest plot of the estimated effects of sarcopenia on osteoporosis risk. (B) Forest plot of the estimated effects for each SD increase in RASM on osteoporosis risk. (C) Forest plot of the estimated effects of osteoporosis on sarcopenia risk. RASM = relative appendicular skeletal muscle mass, SD = standard deviation.

A pooled analysis of 38 studies involving 224,321 participants revealed that sarcopenia was significantly associated with the risk of osteoporosis (OR, 3.06; 95% CI, 2.30 to 4.08; Pheterogeneity < .001, *I*^2^ = 93.7%) (Fig. 2A). Furthermore, this pooled analysis of 7 studies with 171,514 participants showed that each SD increase in RASM significantly decreased osteoporosis risk (OR, 0.65; 95% CI, 0.56–0.75; *P* = .002, I^2^ = 71.5%) (Fig. 2B). Sensitivity analysis indicated that the overall combined results were stable despite our exclusion of each study iteratively (Figs. S1 and S2, Supplemental Digital Content, http://links.lww.com/MD/H889). The subgroup analyses with respect to sex, study design, sarcopenia definitions, and region also revealed that sarcopenia significantly increased the risk of osteoporosis (Table S2, Supplemental Digital Content, http://links.lww.com/MD/H888). Begg rank correlation test showed that publication bias existed in the studies (Fig. S3A, Supplemental Digital Content, http://links.lww.com/MD/H890); however, the funnel plot became symmetrical after the trim-and-fill method was applied (Fig. S3B, Supplemental Digital Content, http://links.lww.com/MD/H890), and the results of the pooled analysis did not vary significantly. The trend toward an increase in osteoporosis risk with sarcopenia persisted (OR, 2.22; 95% CI, 2.13–2.31) (Fig. S3C, Supplemental Digital Content, http://links.lww.com/MD/H890).

### 3.3. Outcomes regarding the association of osteoporosis to the risk of sarcopenia

A pooled analysis of 17 studies indicated that osteoporosis significantly related to sarcopenia risk (OR, 2.63; 95% CI, 1.98–3.49; Pheterogeneity_ < _.001, *I*^2^ = 71.4%) (Fig. 2C). Sensitivity analysis suggested that the substantial heterogeneity did not alter the stability of the outcome (Fig. S4, Supplemental Digital Content, http://links.lww.com/MD/H891). In the subgroup analyses with respect to sex, study design, sarcopenia definitions, and region, we found that osteoporosis clearly had relation to the risk of sarcopenia (Table S2, Supplemental Digital Content, http://links.lww.com/MD/H888). There was no evidence of publication bias in Begg’s rank correlation test in the studies examining the contributions of osteoporosis (*P* > |*z*| =.405) to increased sarcopenia risk (Fig. S5, Supplemental Digital Content, http://links.lww.com/MD/H892).

## 4. Discussion

Sarcopenia and osteoporosis are highly prevalent in older adults and contribute to various adverse health outcomes.^[70]^ Our pooled analysis aimed to describe the interrelationship between sarcopenia and osteoporosis risk and indicated that sarcopenia significantly associated with the higher osteoporosis risk; intriguingly, people with osteoporosis are more likely to suffer from sarcopenia. In addition, 7 trials with 171,514 participants showed that each SD increase in RASM significantly decreased osteoporosis risk. The above results suggest that sarcopenia and osteoporosis have a highly positive correlation.

Sarcopenia is a pivotal contributing factor to osteoporosis^[1]^; therefore, early prevention of sarcopenia is essential.^[7]^ In a study comprising Finish postmenopausal women, those with sarcopenia had a 12.9-fold risk of osteoporosis compared to those without sarcopenia.^[21]^ In another study, a higher risk of osteoporosis was noted in older women with sarcopenia than in those without sarcopenia (OR, 3.45 [95% CI, 1.52–7.84]).^[58]^ In our pooled analysis of studies regarding the contribution of sarcopenia to osteoporosis, the heterogeneity was substantial, and publication bias existed; however, each of the subgroup analyses demonstrated the credibility of the results and showed that sarcopenia significantly increased the risk of osteoporosis. The results of the sensitivity analysis were stable, although we excluded each study sequentially. After the trim-and-fill method was used, the funnel plot became symmetrical, and the publication bias disappeared. The results of the pooled analysis did not vary significantly, and the tendency of sarcopenia to the higher the risk of osteoporosis persisted, indicating the credibility of the results. Therefore, increasing muscle mass by the early detection and treatment of sarcopenia could be beneficial to reduce the risk of osteoporosis and related adverse events.

Osteoporosis is a systemic condition of the skeleton that is characterized by low bone mass and quality, which can increase the susceptibility to sarcopenia.^[1,2]^ Osteoporosis is related to an increased risk of sarcopenia.^[6]^ However, some studies indicated that people with osteoporosis alone do not have an elevated risk for sarcopenia.^[7,30,34]^ Regarding our pooled analysis of studies on the contribution of osteoporosis to sarcopenia, the sensitivity analysis showed that the results were robust, and the findings remained meaningful. Each pooled subgroup, classified by study design, sex, sarcopenia definition, and region, showed that osteoporosis significantly increased the risk of sarcopenia. Therefore, we suggest that it is possible to prevent sarcopenia by increasing bone mass through osteoporosis treatment.

Sarcopenia and osteoporosis are musculoskeletal conditions that interact with each other, and both are associated with aging, sex, height, smoking, physical activity, lifestyle factors, and similar risk factors, such as blood vitamin D levels, genetics, common etiological pathways, endocrine function, and mechanical factors.^[42,58]^ Therefore, specific growth factors may mediate this phenomenon of bidirectional bone-muscle crosstalk.^[71,72]^

Moreover, sarcopenia and osteoporosis often occur simultaneously in the same individual, and both are closely related to bone fragility, increased risk of falls, fractures, and related complications;^[73]^hence, we recommend that patients undergo bone mineral density examinations and investigations to determine the presence of sarcopenia at the same time. Our pooled analysis reinforces previous findings that suggest that the concept of “osteoporosis-skeletal muscle reduction” should not be ignored.

There are several limitations to our study. First, different types of observational studies, such as cross-sectional studies, case–control studies, and cohort studies, were analyzed, leading to substantial heterogeneity. Second, we had to calculate these values according to the specific numbers of participants because some trials did not provide the data as estimates with 95% CIs. Thus, the accuracy of the results might have been influenced. Third, some trials explored the association between sarcopenia and osteoporosis at the community level. In contrast, others explored the association based on specific population clusters that were grouped according to commonalities such as hip fracture, chronic disease, and other conditions, and these might have generated heterogeneity among the studies and exaggerated the positive findings. Fourth, the methods for adjusting confounding factors varied across different studies, possibly contributing to some uncertainty regarding the estimates. Overall, our findings should be interpreted with caution, and randomized controlled trials with large sample sizes are needed to resolve the uncertainty regarding the association between osteoporosis and sarcopenia.

## 5. Conclusions

Our pooled analysis showed that sarcopenia and osteoporosis have a highly positive correlation; sarcopenia significantly related the risk of osteoporosis, and osteoporosis associated with the higher risk of sarcopenia. These findings highlight the importance of sarcopenia screening for those at risk of osteoporosis, and vice versa. Thus, we suggest that the “osteoporosis-sarcopenia” concept should receive more attention.

## Acknowledgments

We sincerely thank all the patients, their families, investigators, and medical staff for their contributions.

## Author contributions

**Conceptualization:** Qinggang Hao, Sheng Lu, Zhaowei Teng.

**Data curation:** Xiaochao Yu, Shuo Sun, Shaoxiong Zhang, Qinggang Hao, Boheng Zhu, Qing Long, Shujun Li, Yan Lv, Qiaoning Yue, Sheng Lu, Zhaowei Teng.

**Formal analysis:** Xiaochao Yu, Shuo Sun, Boheng Zhu, Sheng Lu, Zhaowei Teng.

**Funding acquisition:** Yirong Teng, Sheng Lu, Zhaowei Teng.

**Investigation:** Xiaochao Yu, Shaoxiong Zhang, Qinggang Hao, Yirong Teng, Qing Long, Shujun Li, Yan Lv, Qiaoning Yue, Sheng Lu, Zhaowei Teng.

**Methodology:** Xiaochao Yu, Shuo Sun, Shaoxiong Zhang, Qinggang Hao, Qing Long, Shujun Li, Yan Lv, Sheng Lu, Zhaowei Teng.

**Project administration:** Yirong Teng, Zhaowei Teng.

**Resources:** Shuo Sun, Shaoxiong Zhang, Qinggang Hao, Boheng Zhu, Yirong Teng, Qiaoning Yue, Sheng Lu, Zhaowei Teng.

**Software:** Xiaochao Yu, Shuo Sun, Zhaowei Teng.

**Supervision:** Xiaochao Yu, Shuo Sun, Zhaowei Teng.

**Validation:** Xiaochao Yu, Qinggang Hao, Boheng Zhu, Zhaowei Teng.

**Visualization:** Xiaochao Yu, Shuo Sun, Qinggang Hao, Boheng Zhu, Zhaowei Teng.

**Writing – original draft:** Xiaochao Yu, Zhaowei Teng.

**Writing – review & editing:** Xiaochao Yu, Qinggang Hao, Sheng Lu, Zhaowei Teng.

## Supplementary Material
