# Supplementary material for: A pooled analysis of the association between sarcopenia and osteoporosis
Source: Medicine (Baltimore). 2022 Nov 18;101(46):e31692. doi: 10.1097/MD.0000000000031692 (PMC9678526; doi:10.1097/MD.0000000000031692)
Supplement: Supplementary file 2 [file medi-101-e31692-s002.pdf]

S2 File. Literature search strategies.

Database: PubMed (from inception to September 1, 2022)

Search strategy:

((((((((("Sarcopenia"[Mesh]) OR "Muscular Atrophy"[Mesh])) OR Sarcopenia[Title/Abstract]) OR loss of muscle mass[Title/Abstract]) OR loss of skeletal muscle mass[Title/Abstract]) OR decrease in muscle mass[Title/Abstract]) OR Muscular Atrophy[Title/Abstract])) AND (((((((("Osteoporosis, Postmenopausal"[Mesh]) OR "Osteoporosis"[Mesh] OR "Bone Diseases, Metabolic"[Mesh])) OR osteoporosis[Title/Abstract]) OR osteopenia[Title/Abstract]) OR low bone mineral density[Title/Abstract]) OR loss of bone mass[Title/Abstract]) OR decrease in bone mass[Title/Abstract])) AND (("Cohort Studies"[Mesh]) OR ("Case-Control Studies"[Mesh]) OR ("Cross-Sectional Studies"[Mesh]) OR ("Observational Studies as Topic"[Mesh]) OR ("Longitudinal Studies"[Mesh]) OR ("Odds Ratio"[Mesh]) OR ("Risk"[Mesh]) OR (odds AND ratio*) OR (relative AND risk) OR ((Cohort Stud*[Title/Abstract]) OR (Case-Control Stud*[Title/Abstract]) OR (Cross-Sectional Stud*[Title/Abstract]) OR (Observational Stud*[Title/Abstract])) OR (Longitudinal Stud*[Title/Abstract]))

Database: Embase (from inception to September 1, 2022)

Search strategy:

Search Number	Query	Results
#1	‘sarcopenia’/exp OR sarcopenia	19653
#2	‘osteoporosis’/exp OR osteoporosis	187444
#3	‘osteopenia’/exp OR ‘osteopenia’	26934
#4	#2 OR #3	197188
#5	cohort AND stud*	1337950
#6	‘case control’ AND stud*	273007

#7	‘cross sectional’ AND stud*	706092
#8	observational AND stud*	447834
#9	(‘longitudinal’/exp OR longitudinal) AND stud*	417695
#10	odds AND ratio*	440332
#11	‘risk’/exp OR risk	4617823
#12	#5 OR #6 OR #7 OR #8 OR #9 OR #10 OR #11	6393242
#13	#1 AND #4 AND #12	1736
