# Supplementary material for: A pooled analysis of the association between sarcopenia and osteoporosis
Source: Medicine (Baltimore). 2022 Nov 18;101(46):e31692. doi: 10.1097/MD.0000000000031692 (PMC9678526; doi:10.1097/MD.0000000000031692)
Supplement: Supplementary file 3 [file medi-101-e31692-s003.pdf]

S1 Table. Characteristics of the 51 eligible studies

Study, year	Age (years)	Gender	Study design	Region	Sample size	OR	LCI	UCI	Interaction	Population	Statistical analysis method	Adjustment factors	Diagnostic criteria for osteoporosis	Diagnostic criteria for sarcopenia
Gillette-Guyonnet, 2000[1]	81.8±1 9	Female	Cross-sectional study	France	129	0.750	0.300	1.840	SP increases OP	Community-based population	Multiple linear regression	Confounding factors	World Health Organization (WHO) diagnostic classification: T-score ≤ -2.5 SDs	Appendicular skeletal muscle mass (ASM, kg)/height^2 (m^2)< 5.45 kg/m^2
Walsh, 2006[2]	17-77	Female	Cross-sectional study	UK	213	12.063	1.592	91.406	OP increases SP	Community-based population	Chi-square test	NA	WHO：T-score below -2.5 SDs	Relative skeletal muscle index (RSMI) (ASM divided by height) below 5.45 kg/m2
Cocker, 2010[3]	≥85	Female and male	NA	UK	167	0.890	0.240	3.320	OP increases SP	Over 85 years of age	Regression analysis	Age and weight	WHO diagnostic classification: T-score ≤ -2.5 SDs	Appendicular lean mass (aLM) divided by the subject's squared height 2 SD
Monaco, 2011[4]	79.7±7. 4	Female	Cross-sectional study	Italy	313	1.800	1.073	3.018	SP increases OP	Older women with hip fracture	Binary logistic regression analysis	Age and interval between fracture and dual-energy X-ray absorptiometry (DXA) scan	WHO diagnostic classification: T-score ≤ -2.5 SDs	RASM (aLM)/height2 (m2)<7.26 kg/m2
Albala, 2012[5]	≥60	Female and male	Cross-sectional study	Chile	741	3.620	2.080	6.330	SP increases OP	Population registered in the Active Life Expectancy,	Multinomial logistic regression analysis	Age and gender	WHO standards	Skeletal muscle mass index (SMI) calculated as ASM/height2 based on sex-

Study, year	Age (years)	Gender	Study design	Region	Sample size	OR	LCI	UCI	Interaction	Population	Statistical analysis method	Adjustment factors	Diagnostic criteria for osteoporosis	Diagnostic criteria for sarcopenia
										Ageing, and Disability Related to Obesity Study				specific lowest 20%
Falutz, 2013 (M)[6]	18-75	Male	NA	Modena	1243	4.954	3.345	7.336	SP increases OP	HIV patients	Chi-square test	NA	c2 analysis-determined differences between proportions of patients with osteoporosis and normal bone mineral density (BMD)	Baumgartner (<7.26 kg/height2 in males)
Falutz, 2013 (W)[6]	15-70	Female	NA	Modena	724	1.479	0.671	3.261	SP increases OP	HIV patients	Chi-square test	NA	c2 analysis-determined differences between proportions of patients with osteoporosis and normal BMD	Baumgartner (<5.45 kg/height2 in females)
Go, 2013[7]	>50	Male	Cross-sectional study	Korea	1397	2.140	0.870	5.310	OP increases SP	Korea National Health and Nutrition Examination Survey (KNHANES) IV (2007-2009) participants	Logistic regression analysis	Age, body mass index (BMI), calcium intake, regular exercise, smoking, alcohol, and education NA	WHO diagnostic classification: T-score ≤ -2.5 SDs	Baumgartner: cutoff point: 6.93 kg/m2

Study, year	Age (years)	Gender	Study design	Region	Sample size	OR	LCI	UCI	Interaction	Population	Statistical analysis method	Adjustment factors	Diagnostic criteria for osteoporosis	Diagnostic criteria for sarcopenia
Miyakoshi, 2013[8]	≥40	Female	Cohort study	Japan	2400	1.560	1.245	1.955	OP increases SP	Orthopedic patients	Chi-square test	NA	WHO diagnostic classification: T-score ≤ -2.5 SDs	RSMI more than 2 SDs below the mean in young women; cutoff point: <5.46 kg/m2 for women
Scott, 2013[9]	60-80	Female and male	Cross-sectional study	USA	582	1.326	0.733	2.400	SP increases OP	Community-dwelling volunteers	Chi-square test	NA	WHO: total hip and/or lumbar spine BMD T-score≤-2.5	Previously reported sex-specific cutoff points for aLM normalized to height from this cohort
Sjöblom, 2013[10]	≥65	Female	Cross-sectional study	Finland	590	9.400	2.100	41.400	SP increases OP	Postmenopausal women	Logistic regression analysis	Age, BMI, physical activity, hormone replacement therapy (HRT), consumption of alcohol and smoking	WHO diagnostic classification: T-score ≤ -2.5 SDs	RASM (aLM)/height2 (m2)<6.3 kg/m2
Verschuere, 2013[11]	59.6±10.7	Male	Cross-sectional study	UK and Belgium	679	3.000<br>0.700	1.600<br>0.500	5.800<br>0.900	SP increases OP<br>Each SD increase decreases OP	Population registered in the European Male Ageing Study (EMAS)	Logistic regression analysis	Age and center	WHO diagnostic classification: T-score ≤ -2.5 SDs	RASM (aLM)/height2 (m2)<7.26 kg/m2

Study, year	Age (years)	Gender	Study design	Region	Sample size	OR	LCI	UCI	Interaction	Population	Statistical analysis method	Adjustment factors	Diagnostic criteria for osteoporosis	Diagnostic criteria for sarcopenia
Huh, 2014 (M)[12]	≥65	Male	Cross-sectional study	Korea	940	3.440	1.730	6.830	SP increases OP	KNHANESI V (2008-2009) participants	Multivariate logistic regression analysis	Relative total fat mass, Homeostatic Model Assessment for Insulin Resistance (HOMA-IR) score, current smoking status, regular exercise, total cholesterol, triglyceride, etc.	WHO diagnostic classification: T-score ≤ -2.5 SDs	RASM <5.04 kg/m2
						0.460	0.327	0.648	Each SD increase decreases OP					
Huh, 2014 (W)[12]		Female			1324	1.760	1.020	3.040	SP increases OP					
						0.809	0.693	0.943	Each SD increase decreases OP					
Kim, 2014[13]	≥65	Male	Cross-sectional study	Korea	765	6.830	1.080	43.410	OP increases SP	KNHANESI V (2008-2009) participants	Multiple logistic regression analysis	Age, regular exercise, family income, total hip BMD, lumbar spine BMD, high-risk drinking, smoking status, total energy intake, protein intake, calcium intake, and serum vitamin D level	WHO diagnostic classification: T-score ≤ -2.5 SDs	ASMI value of 7.04 kg/m^2

Study, year	Age (years)	Gender	Study design	Region	Sample size	OR	LCI	UCI	Interaction	Population	Statistical analysis method	Adjustment factors	Diagnostic criteria for osteoporosis	Diagnostic criteria for sarcopenia
Kim SY, 2014 (M)[14]	≥65	Male	Cross-sectional study	Korea	1308	2.120	1.330	3.370	SP increases OP	KNHANES IV (2008-2010) participants	Logistic analysis	Age, fat mass, calcium intake, vitamin D status, smoking, alcohol consumption, and physical activity	WHO diagnostic classification: T-score ≤ -2.5 SDs	RASM (aLM)/height2 (m2)<6.85 kg/m2
Kim SY, 2014 (W)[14]	≥65	Female			1171	1.150	0.810	1.650	SP increases OP		Logistic analysis			RASM (aLM)/height2 (m^2)<5.96 kg/m2
Albala, 2015[15]	60-99	Female and male	Cross-sectional study	Chile	991	3.124	2.223	4.390	OP increases SP	Community-dwelling population	Chi-square test	NA	WHO standards	Sarcopenia: SMI calculated as ASM/height^2 based on sex-specific lowest 20%
Wang, 2015 (M)[16]	≥65	Male	Cross-sectional study	China	164	1.898	0.910	3.959	SP increases OP	Community-dwelling Chinese elders	Chi-square test	NA	WHO diagnostic classification: T-score ≤ -2.5 SDs	Asian Working Group for Sarcopenia (AWGS) cutoff values for muscle mass measurements (7.0 kg/m^2 for men and 5.7 kg/m^2 for women by using bioimpedance analysis [BIA])
Wang, 2015 (W)[16]		Female			152	0.944	0.480	1.856	SP increases OP					
Chung, 2016[17]	≥50	Female and male	Cross-sectional study	Korea	2344	3.087	2.144	4.443	SP increases OP	KNHANES V (2010) participants	Multivariable logistic regression analysis	Age, sex, household income, current smoking status, alcohol	T-score ≤-2.5	SMI score in the fifth percentile of sex-matched younger (20-40 years of age)

Study, year	Age (years)	Gender	Study design	Region	Sample size	OR	LCI	UCI	Interaction	Population	Statistical analysis method	Adjustment factors	Diagnostic criteria for osteoporosis	Diagnostic criteria for sarcopenia
												consumption, vitamin D, hypertension and dyslipidemia		reference KNHANES V-1 participants; SMI cutoff values: 28.9% for men and 22.4% for women
Maghraoui, 2016[18]	40.9 ± 11.0	Male	Case-control study	Morocc o	67	4.375	1.125	17.019	SP increases OP	Ankylosing spondylitis (AS) patients	Chi-square test	NA	WHO diagnostic classification: T-score ≤ -2.5 SDs	EWGSOP
Hars, 2016[19]	63-67	Female and male	Cohort study	Switzerl and	913	2.39	1.51	3.79	SP increases OP	Community-dwelling population	Multivariat e logistic regression analysis	Sex, age, duration of follow-up, etc.	WHO diagnostic classification: T-score ≤ -2.5 SDs	Baumgartner's definition: 5.45 kg/m2 in women and 7.26 kg/m2 in men
He, 2016[20]	18-97.5	Female and male	Cross-sectional study	China<br><br>USA	17891	0.63	0.59	0.66	Each SD increase decreases OP	Chinese individuals<br><br>African American individuals<br><br>Caucasian individuals	Multivariat e logistic regression analysis	Age, gender, height, weight, race, city, smoking, alcohol drinking, and regular exercise	WHO diagnostic classification	(1) 6.08 and 4.79 kg/m2 for healthy Chinese men and women, respectively; (2) RASM ≤7.26 kg/m2 and RASM ≤5.45 kg/m2 in men and women, respectively, plus either low muscle strength or low physical performance
Hong, 2016 (M)[21]	≥65	Male	Cross-sectional study	Korea	1373	3.89	2.265	6.781	SP increases OP	KNHANES IV (2008-	Multivariat e logistic	Age, exercise habits, alcohol consumption,	WHO diagnostic classification: T-score ≤ -2.5 SDs	Sarcopenia: <7.02 kg/m2

Study, year	Age (years)	Gender	Study design	Region	Sample size	OR	LCI	UCI	Interaction	Population	Statistical analysis method	Adjustment factors	Diagnostic criteria for osteoporosis	Diagnostic criteria for sarcopenia
										2010) participants	regression analysis	smoking habits, vitamin D levels, and nutritional factors		
Hong, 2016 (W)[21]	≥65	Female	Cross-sectional study	Korea	1803	1.868	1.227	2.844	SP increases OP	KNHANES IV (2008-2010) participants	Multivariate logistic regression analysis	Age, exercise habits, alcohol consumption, smoking habits, vitamin D levels, and nutritional factors	WHO diagnostic classification: T-score ≤ -2.5 SDs	Sarcopenia: <5.00 kg/m2 in women
Lee, 2016[22]	≥50	Female and male	Cross-sectional study	Korea	858	6.952	3.418	14.139	SP increases OP	KNHANES IV, V (2008-2011) participants with chronic obstructive pulmonary disease (COPD)	Multivariate logistic regression analysis	Age; gender; height; smoking frequency; blood levels of vitamin D, parathyroid hormone (PTH), and alkaline phosphatase (ALP); forced expiratory volume in 1 second (FEV1, %); and physical inactivity level	WHO diagnostic classification: T-score ≤ -2.5 SDs for osteoporosis	AWGS：ASMI by DXA ≤7.0 kg/m2 for male patients and ≤ 5.4 kg/m2 for female patients
Yoshimura,2016[23]	≥60	Female and male	Cohort study	Japan	1099	2.990	1.460	6.120	OP increases SP	ROAD Ⅱ	Logistic regression analysis	Age and FM	WHO: T-score <-2.5	AWGS Criteria

Study, year	Age (years)	Gender	Study design	Region	Sample size	OR	LCI	UCI	Interaction	Population	Statistical analysis method	Adjustment factors	Diagnostic criteria for osteoporosis	Diagnostic criteria for sarcopenia
Monaco, 2017[24]	79.6±7.4	Female	Cross-sectional study	Italy	653	1.220	0.840	1.760	SP increases OP	Female hip fracture patients	Binary logistic regression analysis	Age and FM	WHO: T-score <-2.5	aLM/height2 < 2 SDs below the mean of the young reference group
Frisoli, 2017[25]	78.91 ± 6.97	Female and male	Cross-sectional study	Brazil	282	3.370	1.892	6.001	OP increases SP	Older outpatients at the Cardiology Division of Federal University	Logistic regression analysis	Age and gender	WHO criteria	EWGSOP criteria (low GS or low gait speed plus low appendicular muscle mass)
Frisoli, 2017 (M)[26]	NA (older adults)	Male	Cross-sectional study	Brazil	21599	9.759	9.126	10.437	SP increases OP	SARCopenia and OSteoporosis in Older Adults with Cardiovascular Diseases (SARCOS) study participants	Chi-square test	NA	Osteoporosis was defined as BMD t-score ≤ - 2.5 SDs (lumbar spine or proximal femur)	Sarcopenia: EWGSOP criteria (weakness or low gait speed plus low appendicular muscle mass)
Frisoli, 2017 (W)[26]	NA (older adults)	Female	Cross-sectional study	Brazil	116	2.895	1.048	7.993	SP increases OP	SARCOS study participants	Chi-square test	NA	Osteoporosis was defined as BMD t-score ≤ - 2.5 SDs (lumbar spine or proximal femur)	Sarcopenia: EWGSOP criteria (weakness or low gait speed plus low appendicular muscle mass)
Kim KM, 2017 (M)[27]	≥65	Male	Cross-sectional study	Korea	711	0.420	0.120	0.760	Each SD increase decreases OP	KNHANES IV (2009) participants	Logistic regression analysis	Age and BMI	Osteoporosis: BMD T-score ≤-2.5 and T-score	RASM, calculated as the ASM adjusted by the squared

Study, year	Age (years)	Gender	Study design	Region	Sample size	OR	LCI	UCI	Interaction	Population	Statistical analysis method	Adjustment factors	Diagnostic criteria for osteoporosis	Diagnostic criteria for sarcopenia
														height (ASM/height2)
Kim KM, 2017 (W)[27]	≥65	Female	Cross-sectional study	Korea	847	0.870	0.610	1.130	Each SD increase decreases OP	KNHANES IV (2009) participants	Logistic regression analysis	Age and BMI	Osteoporosis: BMD T-score ≤-2.5 and T-score	RASM, calculated as the ASM adjusted by the squared height (ASM/height2)
Magdalena, 2017 (PSA)[28]	50-75	Female	Case-control study	Poland	51	3.323	0.920	12.009	SP increases OP	Psoriatic arthritis patients	Chi-square test	NA	WHO diagnostic classification: T-score ≤ -2.5 SDs	Baumgartner et al.: aLM index < 5.45 kg/m2
Lee, 2017 (ACOS)[29]	≥50	Female and male	Cross-sectional study	Korea	110	9.611	1.133	81.544	SP increases OP	KNHANES IV, V (2008-2011) participants with asthma-COPD overlap syndrome (ACOS)	Multivariate logistic regression analysis	Age; gender; height; smoking frequency; blood levels of vitamin D, PTH, and ALP; FEV1 (%); and physical inactivity level	WHO diagnostic classification: T-score ≤ -2.5 SDs	AWGS; sarcopenia: ASMI by DXA ≤7.0 kg/m2 for male patients and ≤ 5.4 kg/m2 for female patients
Lee, 2017 (COPD)[29]					748	5.476	2.866	10.464	SP increases OP					
Lee, 2017 (Asthma)[29]					89	0.433	0.030	6.221	SP increases OP					
Locquet, 2017[30]	≥65	Female	Cross-sectional study	Belgium	126	3.039	1.230	7.509	SP increases OP	SarcoPhAge	Chi-square test	NA	WHO diagnostic classification: T-score ≤ -2.5 SDs	EWGSOP criteria
Monaco, 2018[31]	81.4±7.6	Male	Cross-sectional study	Italy	80	4.830	1.170	19.980	SP increases OP	Male hip fracture patients	Binary logistic regression analysis	Age and FM	Osteoporosis: T-score < -2.5 at either	Foundation for the National Institutes of Health (FNIH)

Study, year	Age (years)	Gender	Study design	Region	Sample size	OR	LCI	UCI	Interaction	Population	Statistical analysis method	Adjustment factors	Diagnostic criteria for osteoporosis	Diagnostic criteria for sarcopenia
													the femoral neck or total hip	criteria for men: aLM < 19.75 kg or aLM/BMI ratio <0.789
Frisoli, 2018 (M)[32]	≥65	Male	Cross-sectional study	Brazil	141	2.984<br>2.930	1.144<br>1.044	7.809<br>8.237	SP increases OP<br>OP increases SP	Older adult outpatients at an outpatient geriatric cardiology clinic	Binomial logistic regression analyses	Age, diabetes mellitus, falls in the last 6 months, etal	WHO's criteria: T-score ≤ -2.5 SDs	EWGSOP criteria
Frisoli, 2018 (W)[32]	≥65	Female	Cross-sectional study	Brasil	191	2.093<br>2.081	0.962<br>0.787	3.714<br>5.500	SP increases OP<br>OP increases SP	Older adult outpatients from an outpatient geriatric cardiology clinic	Binomial logistic regression analyses	age, smoking history, diabetes mellitus, , etal	WHO criteria: BMD T-score ≤ -2.5 SDs at lumbar spine, femur neck, and total femur	EWGSOP Criteria
Hayashi, 2018[33]	Controls: 68 (median)<br>Cases: 72 (median)	Female and male	Case-control study	Japan	112	3.508	1.074	11.456	OP increases SP	Hepatocellular carcinoma patients	Multivariate logistic regression analysis	Sex and age	WHO diagnostic classification: T-score ≤ -2.5 SDs	Skeletal muscle reduction: SMI cutoff for Asians (men <7.0 kg/m2, women <5.4 kg/m2)
Hayashi, 2018[34]	Median: 65	Female and male	Chronic liver disease	Japan	112	6.160	1.100	34.600	SP increases OP	Patients with chronic liver disease	Multivariate logistic analysis	NA	WHO diagnostic classification: T-score ≤ -2.5 SDs	Working Group for the Creation of Sarcopenia Assessment Criteria in the Japan Society of Hepatology

Study, year	Age (years)	Gender	Study design	Region	Sample size	OR	LCI	UCI	Interaction	Population	Statistical analysis method	Adjustment factors	Diagnostic criteria for osteoporosis	Diagnostic criteria for sarcopenia
														cutoffs: 7.0 kg/m2 for males and 5.7 kg/m2 for females
Yoo, 2018[35]	≥ 30	Male	Cross-sectional study	Korea	6104	3.850	2.250	6.580	SP increases OP	KNHANES (2008-2011) participants	Multivariate logistic regression analysis	Age, BMI, comorbidities, SMI, physical activity, alcohol consumption, smoking status, calcium intake, income level, and education level	Osteoporosis WHO criteria: T-score of ≤- 2.5 in men aged ≥ 50 years; Z score ≤ − 2.0 for men aged < 50 years	SMI< 6.58 kg/m2
Du, 2019 (M)[36]	>65	Male	Cross-sectional study	China	213	4.210	1.320	13.250	SP increases OP	Community-dwelling older adults	Logistic regression analysis	Age	Osteoporosis: T-score <-2.5 for either site	Cutoff values from previous study: 6.66 kg/m2 for males + low muscle strength+ poor physical performance
Du, 2019 (W)[36]	>65	Female	Cross-sectional study	China	418	9.320	2.540	32.170	SP increases OP	Community-dwelling older adults	Logistic regression analysis	Age	Osteoporosis: T-score < -2.5 for either site	Cutoff values from previous study: 6.66 kg/m2 for males + low muscle strength+ poor physical performance

Study, year	Age (years)	Gender	Study design	Region	Sample size	OR	LCI	UCI	Interaction	Population	Statistical analysis method	Adjustment factors	Diagnostic criteria for osteoporosis	Diagnostic criteria for sarcopenia
Kobayashi, 2019[37]	≥60	Female and male	Longitudinal study	Japan	124	3.125	1.068	9.091	OP increases SP	Community residents	Multivariate logistic analysis	NA	Osteoporosis: BMD with a percent of the young adult mean (%YAM) <70%	Presarcopenia in Japanese people: ASMI <7.0 kg/m2 and <5.8 kg/m2 in males and females by BIA
Lima, 2019[38]	68.3 ± 6.3 years	Female	Cross-sectional study	Brazil	234	2.515	1.046	6.047	SP increases OP	Elderly women	Logistic regression analysis	Age and BMI	Osteoporosis: BMD value (hip or spine) 2.5 SDs below the mean for a young adult reference population	EWGSOP criteria
Locquet, 2019[39]	75.5±5.4 years	Female and male	Cross-sectional study	Belgium	232	4.750	1.160	19.410	SP increases OP	SarcoPhAge study participants	Chi-square test	Age, sex, BMI, number of comorbidities, prescribed medicines, nutritional and cognitive status, and physical activity level	WHO diagnostic classification: T-score ≤ -2.5 SDs	EWGSOP criteria
Papageorgiou, 2019[40]	60.3 ± 5.5 years	Female and male	Cohort study	UK	149122	0.540	0.450	0.650	Each SD increase decreases OP	UK Biobank cohort	Multiple regression analysis	Age, ethnicity, self-reported smoking and alcohol use, physical activity, use of HRT, and self-reported diagnosed	WHO diagnostic classification: T-score ≤ -2.5 SDs	EWGSOP criteria

Study, year	Age (years)	Gender	Study design	Region	Sample size	OR	LCI	UCI	Interaction	Population	Statistical analysis method	Adjustment factors	Diagnostic criteria for osteoporosis	Diagnostic criteria for sarcopenia
												cancer and diabetes as covariates		
Reiss, 2019[41]	≥70	Female and male	Cross-sectional study	Austria	148	8.710	2.870	26.420	SP increases OP	Geriatric inpatients with sarcopenia	Mantel-Haenszel method of weighted odds ratios	Age and gender	WHO diagnostic classification: T-score ≤ -2.5 SDs	EWGSOP criteria
Saeki, 2019[42]	70.5 (median)	Female and male	Cross-sectional study	Japan	142	5.722	2.179	15.030	SP increases OP	Patients with liver cirrhosis	Multiple logistic regression analysis	NA	WHO diagnostic classification: T-score ≤ -2.5 SDs	Japan Society of Hepatology (JSH) criteria, AWGS criteria
Taniguchi, 2019[43]	≥65	Female	Cross-sectional study	Japan	265	2.560	1.330	4.910	OP increases SP	Community-dwelling older women	Logistic regression analysis	Age, GS, usual walking speed, number of prescribed medicines, exercise habits, and fall history	By interview of the participants	ASMI<5.7 kg/m2
Monaco, 2020[44]	79.7 (7.2)	Female	Cross-sectional study	Italy	350	3.205	1.739	5.907	SP increases OP	350 women with subacute hip fracture	Chi-square test	NA	LBMD with a femoral T-score <-2.5	Low muscle mass: aLM <15.02 kg
Kirk, 2020[45]	≥65	Female and male	Cross-sectional study	Australia	484	2.885	1.155	7.204	OP increases SP	Community-dwelling older adults	Logistic regression analysis	Age, sex and vitamin D levels	T-score≤− 2.5	Sarcopenia: EWGSOP2

Study, year	Age (years)	Gender	Study design	Region	Sample size	OR	LCI	UCI	Interaction	Population	Statistical analysis method	Adjustment factors	Diagnostic criteria for osteoporosis	Diagnostic criteria for sarcopenia
Lera,2020[46]	≥60	female and male	Cohort study	Chile	430	2.800	1.200	6.600	SP increase OP	community-dwelling people 60 years and older	Logistic regression models	age, sex, nutritional state, and lean mass/fat mass ratio	WHO: T-score ≤ -2.5	EWGSOP
Nielsen,2020[47]	63–93	female and male	Cross-sectional study	Denmark	529	7.300	2.300	22.800	SP increase OP	population-based 65+ years older	Fischer Exact test/ Chi-Squared test	NA	WHO: T-score ≤ -2.5	EWGSOP2
Fanny, 2020[48]	>65	Female	Cross-sectional study	UK	396283	4.400	3.560	5.460	OP increases SP	White European participants	Binary logistic regression analysis	Age, sex, deprivation and educational attainment	NA	Sarcopenia: low GS plus low muscle mass using the current EWGSOP2 classification and cutoff points
Yu, 2020[49]	≥60	Female and male	Cross-sectional study	China	658	1.129	0.629	2.025	OP increases SP	Suburban-dwelling participants	Logistic regression analysis	Age, gender, educational level, smoking and drinking habits	WHO diagnostic classification: T-score ≤ -2.5 SDs	AWGS criteria
Lee,2021[50]	≥65	female and male	Cross-sectional study	Korea	3077	2.258	1.584	3.218	SP increase OP	KNHANES (2008–2011) conducted by the Korean Centers for Disease Control and Prevention	multivariate logistic regression analysis	all covariates, such as physical examinations, exercise, and nutrient intake	WHO criteria	AWGS

Study, year	Age (years)	Gender	Study design	Region	Sample size	OR	LCI	UCI	Interaction	Population	Statistical analysis method	Adjustment factors	Diagnostic criteria for osteoporosis	Diagnostic criteria for sarcopenia
Fanny,2021[51]	37-70	female	prospective study	UK	168682	1.660	1.330	2.080	SP increase OP	general population to be part of UK Biobank	Cox-proportional hazard models	socio-demographic, lifestyle and health-related factors, and morbidity count	based on dual-energy X-ray absorptiometry (DXA) scan or women >75 years that experienced a fragility fracture	EWGSOP2
Saeki,2021[52]	68.0 (56.5–73.0)	female and male	Observational	Japan	117	4.126	1.280	13.297	SP increase OP	patients with primary biliary cholangitis	multiple logistic regression analysis	NA	WHO: T-score ⩽ -2.5	The SMI cutoff values for low muscle-mass diagnosis were 7.0 kg/m2 for men and 5.7 kg/m2 for women
Saeki,2021[52]	68.0 (56.5–73.0)	female and male	Observational	Japan	117	3.420	1.057	11.067	OP increase SP	patients with primary biliary cholangitis	multiple logistic regression analysis	NA	WHO: T-score ⩽ -2.5	The SMI cutoff values for low muscle-mass diagnosis were 7.0 kg/m2 for men and 5.7 kg/m2 for women
Tan,2021[53]	⩾18	female and male	and Clinical trials	Other	156	8.440	1.100	64.880	SP increase OP	patients with Parkinson's Disease	Multiple logistic regressions	age, gender, and body mass index as covariates	WHO: T-score ⩽ -2.5	EWGSOP2
Monaco,2022[54]	79.7 ±7.6	female	cross-sectional study	Italy	262	2.300	1.270	4.140	SP increase OP	Women with subacute hip fracture were	Binary logistic regression analysis	age, body fat percentage and time interval	femoral bone mineral density lower than 2.5 standard	EWGSOP2

Study, year	Age (years)	Gender	Study design	Region	Sample size	OR	LCI	UCI	Interaction	Population	Statistical analysis method	Adjustment factors	Diagnostic criteria for osteoporosis	Diagnostic criteria for sarcopenia
										surgically treated in local rehabilitation hospital		between fracture and DXA scan.	deviations below the mean of the young reference population.	
Pan,2022[55]	65.0 ± 9.8	female	cross-sectional study	China	192	4.079	1.440	11.559	SP increase OP	Participants underwent type 2 diabetes mellitus evaluation or treatment at the Second Affiliated Hospital of Wenzhou Medical University and Yuying Children's Hospital	Multivariate regressions	age, diabetes duration, systolic blood pressure, diastolic blood pressure, smoking, alcohol consumption, TG, TC, HDL-C, LDL-C, albumin, creatinine, uric, HbA1c, FBG	WHO criteria	Customized criteria:ASM/height <7.87 kg/m2 in men or <5.94 kg/m2 in women
Pan,2022[55]	67.6 ± 8.8	male	cross-sectional study	China	225	6.036	2.389	15.325	SP increase OP	Participants underwent type 2 diabetes mellitus evaluation or treatment at the Second Affiliated Hospital of Wenzhou	Multivariate regressions	age, diabetes duration, systolic blood pressure, diastolic blood pressure, smoking, alcohol consumption, TG, TC, HDL-C, LDL-C, albumin,	WHO criteria	Customized criteria:ASM/height <7.87 kg/m2 in men or <5.94 kg/m2 in women

Study, year	Age (years)	Gender	Study design	Region	Sample size	OR	LCI	UCI	Interaction	Population	Statistical analysis method	Adjustment factors	Diagnostic criteria for osteoporosis	Diagnostic criteria for sarcopenia
										Medical University and Yuying Children's Hospital		creatinine, uric, HbA1c, FBG		
Xing,2022[56]	68.8 ± 6.5	female and male	cross-sectional study	China	158	2.520	1.130	5.370	OP increase SP	patients aged >60 years with hypertension who visited the Tianjin Port Hospital in China	Multivariable logistic regressions	NA	WHO criteria	AWGS

NA: not available; SP increases OP: sarcopenia is associated with a higher risk of osteoporosis; OP increases SP: osteoporosis is associated with a higher risk of sarcopenia; OR: estimate of the risk; LCI: low limit of 95% confidence interval; UCI: upper limit of 95% confidence interval

References

[1]. Gillette-Guyonnet S, Nourhashemi F, Lauque S, Grandjean H, Vellas B. Body composition and osteoporosis in elderly women. Gerontology. 2000;46(4):189-93.

[2]. Walsh MC, Hunter GR, Livingstone MB. Sarcopenia in premenopausal and postmenopausal women with osteopenia, osteoporosis and normal bone mineral density. Osteoporosis international : a journal established as result of cooperation between the European Foundation for Osteoporosis and the National Osteoporosis Foundation of the USA. 2006;17(1):61-7.

[3]. Cocker M, Francis R, Narici M, Birrell F. Sarcopaenia is highly prevalent in the very elderly and predicts mortality in males. Rheumatology. 2010;49:i62-i3.

[4]. Di Monaco M, Vallero F, Di Monaco R, Tappero R. Prevalence of sarcopenia and its association with osteoporosis in 313 older women following a hip fracture. Archives of gerontology and geriatrics. 2011;52(1):71-4.

[5]. Albala C, Lera L, Sanchez H, Angel B, Fuentes A, Arroyo P. Sarcopenia is more important than vitamin D deficiency as determinant of osteoporosis in chilean elders. Osteoporosis International. 2012;23:S343-S4.

[6]. Falutz J, Rosenthall L, Guaraldi G. Association of osteoporosis and sarcopenia in treated HIV patients. Antiviral Therapy. 2013;18:A17.

[7]. Go SW, Cha YH, Lee JA, Park HS. Association between Sarcopenia, Bone Density, and Health-Related Quality of Life in Korean Men. Korean journal of family medicine. 2013;34(4):281-8.

[8]. Miyakoshi N, Hongo M, Mizutani Y, Shimada Y. Prevalence of sarcopenia in Japanese women with osteopenia and osteoporosis. Journal of bone and mineral metabolism. 2013;31(5):556-61.

[9]. Scott D, Aitken D, Ebeling PR, Sanders K, Hayes A, Jones G. 'Sarco-osteoporosis': The prevalence and functional outcomes of comorbid sarcopenia and osteoporosis in community-dwelling older adults. Osteoporosis International. 2013;24:S563.

[10]. Sjoblom S, Suuronen J, Rikkonen T, Honkanen R, Kroger H, Sirola J. Relationship between postmenopausal osteoporosis and the components of clinical sarcopenia. Maturitas. 2013;75(2):175-80.

[11]. Verschueren S, Gielen E, O'neill TW, et al. Sarcopenia and its relationship with bone mineral density in middle-aged and elderly European men. Osteoporosis international : a journal established as result of cooperation between the European Foundation for Osteoporosis and the National Osteoporosis Foundation of the USA. 2013;24(1):87-98.

[12]. Huh JH, Song MK, Park KH, et al. Gender-specific pleiotropic bone-muscle relationship in the elderly from a nationwide survey (KNHANES IV). Osteoporosis International. 2014;25(3):1053-61.

[13]. Kim JE, Lee YH, Huh JH, Kang DR, Rhee Y, Lim SK. Early-stage chronic kidney disease, insulin resistance, and osteoporosis as risk factors of sarcopenia in aged population: the fourth Korea National Health and Nutrition Examination Survey (KNHANES IV), 2008-2009. Osteoporosis international : a journal established as result of cooperation between the European Foundation for Osteoporosis and the National Osteoporosis Foundation of the USA. 2014;25(9):2189-98.

[14]. Kim S, Won CW, Kim BS, Choi HR, Moon MY. The association between the low muscle mass and osteoporosis in elderly Korean people. Journal of Korean medical science. 2014;29(7):995-1000.

[15]. Albala C, Lera L, Sanchez H, et al. Osteoporosis, sarcopenia and fractures in chilean older people. Osteoporosis International. 2015;26(1):S332.

[16]. Wang YJ, Wang Y, Zhan JK, et al. Sarco-osteoporosis: Prevalence and association with frailty in Chinese community-dwelling older adults. International Journal of Endocrinology. 2015;2015.

[17]. Chung SM, Hyun MH, Lee E, Seo HS. Novel effects of sarcopenic osteoarthritis on metabolic syndrome, insulin resistance, osteoporosis, and bone fracture: the national survey. Osteoporosis international : a journal established as result of cooperation between the European Foundation for Osteoporosis and the National Osteoporosis Foundation of the USA. 2016;27(8):2447-57.

[18]. El Maghraoui A, Ebo'o FB, Sadni S, Majjad A, Hamza T, Mounach A. Is there a relation between pre-sarcopenia, sarcopenia, cachexia and osteoporosis in patients with ankylosing spondylitis? BMC musculoskeletal disorders. 2016;17:268.

[19]. Hars M, Biver E, Chevalley T, et al. Low Lean Mass Predicts Incident Fractures Independently From FRAX: a Prospective Cohort Study of Rece nt Retirees. Journal of bone and mineral research : the official journal of the American Society for Bone and Mineral Research. 2016;31(11):2048-56.

[20]. He H, Liu Y, Tian Q, Papasian CJ, Hu T, Deng HW. Relationship of sarcopenia and body composition with osteoporosis. Osteoporosis international : a journal established as result of cooperation between the European Foundation for Osteoporosis and the National Osteoporosis Foundation of the USA. 2016;27(2):473-82.

[21]. Hong S, Choi WH. The effects of sarcopenia and obesity on femur neck bone mineral density in elderly Korean men and women. Osteoporosis and sarcopenia. 2016;2(2):103-9.

[22]. Lee DW, Choi EY. Sarcopenia as an Independent Risk Factor for Decreased BMD in COPD Patients: Korean National Health and Nutrition Examination Surveys IV and V (2008-2011). PloS one. 2016;11(10):e0164303.

[23]. Yoshimura N, Muraki S, Oka H, et al. Is osteoporosis a predictor for future sarcopenia or vice versa? Four-year observations between the second and third ROAD study surveys. Osteoporosis international : a journal established as result of cooperation between the European Foundation for Osteoporosis and the National Osteoporosis Foundation of the USA. 2016;28(1):189-99.

[24]. Di Monaco M, Castiglioni C, Di Monaco R, Tappero R. Association between low lean mass and low bone mineral density in 653 women with hip fracture: does the definition of low lean mass matter? Aging clinical and experimental research. 2017;29(6):1271-6.

[25]. Frisoli A, Martim F, Borges J, Carvalho A, Chaves P. Association of Osteosarcopenia, Sarcopenia EWGSOP alone and osteoporosis alone with mobility in older adults: Data from sarcos study. Journal of the American Geriatrics Society. 2017;65:S49.

[26]. Frisoli A, Martin F, Ingham S, Carvalho AC. Body composition phenotype of osteosarcopenia, osteoporosis and sarcopenia: SARCOS study. Journal of Bone and Mineral Research. 2017;31.

[27]. Kim KM, Lee EY, Lim S, Jang HC, Kim CO. Favorable effects of skeletal muscle on bone are distinguished according to gender and skeletal sites. Osteoporosis and sarcopenia. 2017;3(1):32-6.

[28]. Krajewska-Wlodarczyk M, Owczarczyk-Saczonek A, Placek W. Changes in body composition and bone mineral density in postmenopausal women with psoriatic arthritis. Reumatologia. 2017;55(5):215-21.

[29]. Lee DW, Jin HJ, Shin KC, Chung JH, Lee HW, Lee KH. Presence of sarcopenia in asthma-COPD overlap syndrome may be a risk factor for decreased bone-mineral density, unlike asthma: Korean National Health and Nutrition Examination Survey (KNHANES) IV and V (2008-2011). International journal of chronic obstructive pulmonary disease. 2017;12:2355-62.

[30]. Locquet M, Beaudart C, Reginster JY, et al. Prevalence of Concomitant Bone and Muscle Wasting in Elderly Women from the SarcoPhAge Cohort: Preliminary Results. The Journal of frailty & aging. 2017;6(1):18-23.

[31]. Di Monaco M, Castiglioni C, Milano E, Massazza G. Is there a definition of low lean mass that captures the associated low bone mineral density? A cross-sectional study of 80 men with hip fracture. Aging clinical and experimental research. 2018;30(12):1429-35.

[32]. Frisoli A, Jr., Martin FG, Carvalho ACC, Borges J, Paes AT, Ingham SJM. Sex effects on the association between sarcopenia EWGSOP and osteoporosis in outpatient older adults: data from the SARCOS study. Archives of endocrinology and metabolism. 2018;62(6):615-22.

[33]. Hayashi F, Kaibori M, Sakaguchi T, et al. Loss of skeletal muscle mass in patients with chronic liver disease is related to decrease in bone mineral density and exercise tolerance. Hepatology research : the official journal of the Japan Society of Hepatology. 2018;48(5):345-54.

[34]. Hayashi M, Abe K, Fujita M, Okai K, Takahashi A, Ohira H. Association between sarcopenia and osteoporosis in chronic liver disease. Hepatology research : the official journal of the Japan Society of Hepatology. 2018;48(11):893-904.

[35]. Yoo JE, Park HS. Prevalence and associated risk factors for osteoporosis in Korean men. Archives of osteoporosis. 2018;13(1):88.

[36]. Du Y, Wang X, Xie H, et al. Sex differences in the prevalence and adverse outcomes of sarcopenia and sarcopenic obesity in community dwelling elderly in East China using the AWGS criteria. BMC endocrine disorders. 2019;19(1):109.

[37].Kobayashi K, Ando K, Tsushima M, et al. Predictors of presarcopenia in community-dwelling older adults: A 5-year longitudinal study. Modern rheumatology. 2019;29(6):1053-8.

[38]. Lima RM, De Oliveira RJ, Raposo R, Neri SGR, Gadelha AB. Stages of sarcopenia, bone mineral density, and the prevalence of osteoporosis in older women. Archives of osteoporosis. 2019;14(1):38.

[39]. Locquet M, Beaudart C, Reginster JY, Bruyère O. Association Between the Decline in Muscle Health and the Decline in Bone Health in Older Individuals from the SarcoPhAge Cohort. Calcified tissue international. 2019;104(3):273-84.

[40]. Papageorgiou M, Sathyapalan T, Schutte R. Muscle mass measures and incident osteoporosis in a large cohort of postmenopausal women. Journal of cachexia, sarcopenia and muscle. 2019;10(1):131-9.

[41]. Reiss J, Iglseder B, Alzner R, et al. Sarcopenia and osteoporosis are interrelated in geriatric inpatients. Zeitschrift fur Gerontologie und Geriatrie. 2019;52(7):688-93.

[42]. Saeki C, Takano K, Oikawa T, et al. Comparative assessment of sarcopenia using the JSH, AWGS, and EWGSOP2 criteria and the relationship between sarcopenia, osteoporosis, and osteosarcopenia in patients with liver cirrhosis. BMC musculoskeletal disorders. 2019;20(1):615.

[43]. Taniguchi Y, Makizako H, Kiyama R, et al. The Association between Osteoporosis and Grip Strength and Skeletal Muscle Mass in Community-Dwelling Older Women. International journal of environmental research and public health. 2019;16(7).

[44]. Di Monaco M, Castiglioni C, Bardesono F, Milano E, Massazza G. Sarcopenia, osteoporosis and the burden of prevalent vertebral fractures: a cross-sectional study of 350 women with hip fracture. European journal of physical and rehabilitation medicine. 2020.

[45]. Kirk B, Phu S, Brennan-Olsen SL, Bani Hassan E, Duque G. Associations between osteoporosis, the severity of sarcopenia and fragility fractures in community-dwelling older adults. European Geriatric Medicine. 2020.

[46]. Lera L, Angel B, Márquez C, Saguez R, Albala C. Software for the Diagnosis of Sarcopenia in Community-Dwelling Older Adults: Design and Validation Study. JMIR medical informatics. 2020;8(4):e13657.

[47]. Nielsen BR, Andersen HE, Haddock B, Hovind P, Schwarz P, Suetta C. Prevalence of muscle dysfunction concomitant with osteoporosis in a home-dwelling Danish population aged 65–93 years - The Copenhagen Sarcopenia Study. Experimental Gerontology. 2020;138.

[48]. Petermann-Rocha F, Chen M, Gray SR, Ho FK, Pell JP, Celis-Morales C. Factors associated with sarcopenia: A cross-sectional analysis using UK Biobank. Maturitas. 2020;133:60-7.

[49]. Yu X, Hou L, Guo J, et al. Combined Effect of Osteoporosis and Poor Dynamic Balance on the Incidence of Sarcopenia in Elderly Chinese Community Suburban-Dwelling Individuals. Journal of Nutrition, Health and Aging. 2020;24(1):71-7.

[50]. Lee DY, Shin S. Association of Sarcopenia with Osteopenia and Osteoporosis in Community-Dwelling Older Korean Adults: A Cross-Sectional Study. Journal of clinical medicine. 2021;11(1).

[51]. Petermann-Rocha F, Ferguson LD, Gray SR, et al. Association of sarcopenia with incident osteoporosis: a prospective study of 168,682 UK biobank participants. Journal of cachexia, sarcopenia and muscle. 2021;12(5):1179-88.

[52]. Saeki C, Oikawa T, Kanai T, et al. Relationship between osteoporosis, sarcopenia, vertebral fracture, and osteosarcopenia in patients with primary biliary cholangitis. European journal of gastroenterology & hepatology. 2020.

[53]. Tan YJ, Lim SY, Yong VW, et al. Osteoporosis in Parkinson's Disease: Relevance of Distal Radius Dual-Energy X-Ray Absorptiometry (DXA) and Sarcopenia. Journal of clinical densitometry : the official journal of the International Society for Clinical Densitometry. 2020.

[54]. M DIM, Castiglioni C, Bardesono F, Freiburger M, Milano E, Massazza G. Is sarcopenia associated with osteoporosis? A cross-sectional study of 262 women with hip fracture. European journal of physical and rehabilitation medicine. 2022;58(4):638-45.

[55]. Pan Y, Xu J. Association between muscle mass, bone mineral density and osteoporosis in type 2 diabetes. Journal of Diabetes Investigation. 2022;13(2):351-8.

[56]. Xing E, Wan C. Prevalence of and factors associated with sarcopenia among elderly individuals with hypertension. The Journal of international medical research. 2022;50(7):3000605221110490.

S3 Table. Methodological quality of studies included in the final analysis based on STROBE statement checklists

No.	Study	Title and abstract				Introduction				Methods				Results				Discussion					Other information
		1	2	3	4	5	6	7	8	9	10	11	12	13	14	15	16	17	18	19	20	21	22
1	Gillette-Guyonnet,2000	yes	yes	yes	yes	yes	yes	yes	yes	unclear	no	yes	yes	yes	yes	yes	yes	yes	yes	No	yes	yes	no
2	Walsh,2006	yes	yes	yes	yes	yes	yes	yes	yes	no	no	yes	yes	yes	yes	yes	yes	yes	yes	yes	yes	yes	yes
3	Cocker,2010	yes	no	no	no	no	no	no	no	no	no	no	no	no	no	no	no	no	no	no	no	no	no
4	Monaco,2011	yes	yes	yes	yes	yes	yes	yes	yes	yes	no	yes	yes	yes	yes	yes	yes	yes	yes	yes	yes	yes	no
5	Albala,2012	yes	no	no	no	no	no	no	no	no	no	no	no	no	no	no	no	no	no	no	no	no	no
6	Falutz,2013	yes	no	no	no	no	no	no	no	no	no	no	no	no	no	no	no	no	no	no	no	no	no
7	Go,2013	yes	yes	yes	yes	yes	yes	yes	yes	unclear	unclear	yes	yes	yes	yes	yes	yes	yes	yes	yes	yes	yes	no
8	Miyakoshi,2013	unclear	yes	yes	no	no	yes	yes	yes	no	no	yes	yes	no	yes	yes	no	no	yes	yes	yes	yes	no
9	Scott,2013	yes	no	no	no	no	no	no	no	no	no	no	no	no	no	no	no	no	no	no	no	no	no
10	Sjöblom,2013	unclear	yes	yes	yes	yes	yes	yes	yes	yes	no	yes	yes	yes	yes	yes	yes	yes	yes	yes	yes	yes	yes
11	Verschueren,2012	unclear	yes	yes	no	no	yes	yes	yes	yes	no	yes	yes	no	yes	yes	yes	yes	yes	yes	yes	yes	yes
12	Huh,2014	unclear	yes	yes	yes	yes	yes	yes	yes	no	no	yes	yes	no	yes	yes	yes	yes	yes	yes	yes	yes	yes
13	Kim,2014	yes	yes	yes	yes	yes	yes	yes	yes	no	no	yes	yes	no	no	yes	yes	yes	yes	yes	yes	yes	yes
14	Kim SY,2014	no	yes	yes	yes	yes	yes	yes	yes	no	no	yes	unclear	yes	yes	yes	yes	no	yes	no	no	yes	no
15	Albala,2015	yes	no	no	no	no	no	no	no	no	no	no	no	no	no	no	no	no	no	no	no	no	no
16	Wang,2015	no	no	yes	yes	yes	yes	yes	yes	no	no	yes	yes	no	yes	yes	yes	yes	yes	yes	yes	yes	yes
17	Chung,2016	unclear	yes	yes	yes	yes	yes	yes	yes	yes	no	yes	unclear	yes	yes	yes	yes	no	yes	yes	yes	yes	yes
18	Maghraoui,2016	no	yes	yes	yes	yes	yes	yes	yes	no	no	yes	unclear	no	yes	yes	yes	no	yes	yes	yes	yes	no
19	Hars,2016	yes	yes	yes	yes	yes	yes	yes	yes	no	no	yes	unclear	no	yes	yes	yes	no	yes	yes	yes	yes	yes
20	He,2016	unclear	yes	yes	yes	yes	yes	yes	yes	no	no	yes	yes	no	yes	yes	yes	yes	yes	yes	yes	yes	yes
21	Hong,2016	unclear	yes	yes	yes	yes	yes	yes	yes	no	no	yes	yes	yes	yes	yes	yes	yes	yes	yes	yes	yes	no
22	Lee,2016	unclear	yes	yes	yes	yes	yes	yes	yes	no	no	yes	yes	yes	yes	yes	yes	yes	yes	yes	yes	yes	yes
23	Yoshimura,2016	yes	yes	yes	yes	yes	yes	yes	yes	yes	no	yes	unclear	yes	yes	yes	yes	no	yes	yes	yes	yes	yes
24	Monaco,2017	unclear	yes	yes	yes	yes	yes	yes	yes	no	no	yes	unclear	yes	yes	yes	yes	no	yes	yes	yes	yes	no
25	Frisoli,2017	yes	no	no	no	no	no	no	no	no	no	no	no	no	no	no	no	no	no	no	no	no	no

26	Frisoli,2017	yes	no	no	no	no	no	no	no	no	no	no	no	no	no	no	no	no	no	no	no	no	no
27	Kim,2017	unclear	yes	yes	yes	yes	yes	yes	yes	no	no	yes	unclear	no	yes	yes	yes	no	yes	yes	yes	yes	no
28	Magdalena,2017	unclear	yes	yes	yes	yes	yes	yes	yes	no	no	yes	unclear	no	yes	yes	no	no	yes	no	yes	yes	no
29	Lee,2017	unclear	yes	yes	yes	yes	yes	yes	yes	no	no	yes	yes	no	yes	yes	yes	yes	yes	yes	yes	yes	no
30	Locquet,2017	yes	yes	yes	yes	yes	yes	yes	yes	no	no	yes	unclear	yes	yes	yes	no	no	yes	yes	yes	yes	no
31	Monaco,2018	yes	yes	yes	yes	yes	yes	yes	yes	no	no	yes	unclear	yes	yes	yes	yes	no	yes	yes	yes	yes	no
32	Frisoli,2018	yes	yes	yes	yes	yes	yes	yes	yes	no	no	yes	unclear	no	yes	yes	yes	no	yes	yes	yes	yes	no
33	Hayashi,2018	unclear	yes	yes	yes	yes	yes	yes	yes	no	no	yes	unclear	yes	no	no	yes	no	yes	yes	yes	yes	no
34	Hayashi,2018	unclear	yes	yes	yes	yes	yes	yes	yes	no	no	yes	unclear	yes	yes	yes	yes	no	yes	yes	yes	yes	no
35	Yoo,2018	unclear	yes	yes	yes	yes	yes	yes	yes	yes	no	yes	unclear	yes	yes	yes	yes	no	yes	yes	yes	yes	no
36	Du,2019	yes	yes	yes	yes	yes	yes	yes	yes	no	no	yes	unclear	yes	yes	yes	yes	no	yes	yes	yes	yes	yes
37	Kobayashi,2019	yes	yes	yes	yes	yes	yes	yes	yes	no	no	yes	unclear	no	yes	yes	yes	no	yes	yes	yes	yes	no
38	Lima,2019	unclear	yes	yes	yes	yes	yes	yes	yes	no	no	yes	unclear	no	yes	yes	yes	no	yes	yes	yes	yes	yes
39	Locquet,2019	unclear	yes	yes	yes	yes	yes	yes	yes	no	no	yes	unclear	yes	yes	yes	yes	no	yes	yes	yes	yes	no
40	Papageorgiou,2019	unclear	yes	yes	yes	yes	yes	yes	yes	no	no	yes	yes	yes	yes	yes	yes	yes	yes	yes	yes	yes	no
41	Reiss,2019	no	yes	yes	yes	yes	yes	yes	yes	no	no	yes	yes	no	yes	yes	yes	yes	yes	yes	yes	yes	yes
42	Saeki,2019	yes	yes	yes	yes	yes	yes	yes	yes	no	no	yes	unclear	no	yes	yes	yes	no	yes	yes	yes	yes	no
43	Taniguchi,2019	yes	yes	yes	yes	yes	yes	yes	yes	no	no	yes	unclear	no	yes	yes	yes	no	yes	yes	yes	yes	yes
44	Monaco,2020	yes	yes	yes	yes	yes	yes	yes	yes	no	no	yes	unclear	yes	yes	yes	yes	no	yes	yes	yes	yes	no
45	Kirk,2020	yes	yes	yes	yes	yes	yes	yes	yes	no	no	yes	unclear	no	yes	yes	yes	no	yes	yes	yes	yes	no
46	Lera.2020	yes	yes	yes	unclear	yes	yes	yes	yes	no	no	yes	yes	yes	yes	yes	yes	no	yes	yes	yes	yes	no
47	Nielsen,2020	yes	yes	yes	yes	yes	yes	yes	yes	yes	no	yes	unclear	no	yes	yes	yes	no	yes	yes	yes	yes	no
48	Fanny,2020	yes	yes	yes	no	no	yes	yes	yes	no	no	yes	unclear	no	yes	yes	yes	no	yes	yes	yes	yes	yes
49	Yu,2020	unclear	yes	yes	yes	yes	yes	yes	yes	no	no	yes	unclear	yes	yes	yes	yes	no	yes	yes	yes	yes	yes
50	Lee,2021	yes	yes	yes	yes	yes	yes	yes	yes	no	no	yes	yes	yes	yes		yes	yes	no	yes	yes	yes	no
51	Fanny,2021	yes	yes	yes	yes	unclear	yes	yes	yes	no	no	yes	yes	yes	yes	yes	yes	yes	yes	yes	yes	yes	unclear
52	Saeki,2021	yes	yes	yes	yes	yes	yes	yes	yes	no	no	yes	unclear	no	yes	yes	yes	no	yes	yes	yes	yes	no
53	Tan,2020	yes	yes	yes	unclear	yes	yes	yes	yes	no	no	yes	unclear	no	yes	yes	yes	no	yes	yes	yes	yes	no

54	Monaco,2022	yes	yes	yes	yes	yes	yes	yes	yes	no	no	yes	yes	yes	yes	yes	yes	yes	yes	yes	yes	yes	no
55	Pan,2022	no	yes	yes	yes	yes	yes	yes	yes	no	yes	no	yes	no	yes	yes	yes	yes	yes	yes	yes	yes	unclear
56	Xing,2022	no	yes	yes	no	yes	yes	yes	yes	no	yes	yes	yes	yes	yes	yes	yes	yes	yes	yes	yes	yes	no

S4 Table. STROBE statement checklists[1].

	Item number	RECOMMENDATION
**TITLE and ABSTRACT**	1	(a) Indicate the study's design with a commonly used term in the title or the abstract<br>(b) Provide in the abstract an informative and balanced summary of what was done and what was found
**INTRODUCTION**		
Background/rationale	2	Explain the scientific background and rationale for the investigation being reported
Objectives	3	State specific objectives, including any prespecified hypotheses
**METHODS**		
Study design	4	Present key elements of study design early in the paper
Setting	5	Describe the setting, locations, and relevant dates, including periods of recruitment, exposure, follow-up, and data collection
Participants	6	(a)Cohort study—Give the eligibility criteria, and the sources and methods of selection of participants. Describe methods of follow-up<br>Case-control study—Give the eligibility criteria, and the sources and methods of case ascertainment and control selection. Give the rationale for the choice of cases and controls<br>Cross-sectional study—Give the eligibility criteria, and the sources and methods of selection of participants<br>(b)Cohort study—For matched studies, give matching criteria and number of exposed and unexposed<br>Case-control study—For matched studies, give matching criteria and the number of controls per case
Variables	7	Clearly define all outcomes, exposures, predictors, potential confounders, and effect modifiers. Give diagnostic criteria, if applicable
	**Item number**	**RECOMMENDATION**

Data sources/measurement	8*	For each variable of interest, give sources of data and details of methods of assessment (measurement)
		Describe comparability of assessment methods if there is more than one group
Bias	9	Describe any efforts to address potential sources of bias
Study size	10	Explain how the study size was arrived at
Quantitative variables	11	Explain how quantitative variables were handled in the analyses. If applicable, describe which groupings were chosen, and why
Statistical methods	12	(a)Describe all statistical methods, including those used to control for confounding
		(b)Describe any methods used to examine subgroups and interactions
		(c)Explain how missing data were addressed
		(d)Cohort study—If applicable, explain how loss to follow-up was addressed
		Case-control study—If applicable, explain how matching of cases and controls was addressed
		Cross-sectional study—If applicable, describe analytical methods taking account of sampling strategy
		(e)Describe any sensitivity analyses
**RESULTS**		
Participants	13*	(a)Report the numbers of individuals at each stage of the study—e.g., numbers potentially eligible, examined for eligibility, confirmed
		eligible, included in the study, completing follow-up, and analysed
		(b)Give reasons for non-participation at each stage
		(c)Consider use of a flow diagram
Descriptive data	14*	(a)Give characteristics of study participants (e.g., demographic, clinical, social) and information on exposures and potential

	**Item number**	**RECOMMENDATION**
		confounders
		(b)Indicate the number of participants with missing data for each variable of interest
		(c)Cohort study—Summarise follow-up time (e.g., average and total amount)
	15*	Cohort study—Report numbers of outcome events or summary measures over time
		Case-control study—Report numbers in each exposure category, or summary measures of exposure
		Cross-sectional study—Report numbers of outcome events or summary measures

Main results	16	(a)Give unadjusted estimates and, if applicable, confounder-adjusted estimates and their precision (e.g., 95% confidence interval).<br>Make clear which confounders were adjusted for and why they were included<br>(b)Report category boundaries when continuous variables were categorized<br>(c)If relevant, consider translating estimates of relative risk into absolute risk for a meaningful time period
Other analyses	17	Report other analyses done—e.g., analyses of subgroups and interactions, and sensitivity analyses
**DISCUSSION**		
Key results	18	Summarise key results with reference to study objectives
Limitations	19	Discuss limitations of the study, taking into account sources of potential bias or imprecision. Discuss both direction and magnitude<br>of any potential bias
Interpretation	20	Give a cautious overall interpretation of results considering objectives, limitations, multiplicity of analyses, results from similar stud<br>ies, and other relevant evidence
Generalisability	21	Discuss the generalisability(external validity) of the study results

	Item number	RECOMMENDATION
**OTHER INFORMATION**		
Funding	22	Give the source of funding and the role of the funders for the present study and, if applicable, for the original study on which the<br>present article is based

References

[1]. Gillette-Guyonnet S, Nourhashemi F, Lauque S, Grandjean H, Vellas B. Body composition and osteoporosis in elderly women. Gerontology. 2000;46(4):189-93.

[2]. Walsh MC, Hunter GR, Livingstone MB. Sarcopenia in premenopausal and postmenopausal women with osteopenia, osteoporosis and normal bone mineral density. Osteoporosis international : a journal established as result of cooperation between the European Foundation for Osteoporosis and the National Osteoporosis Foundation of the USA. 2006;17(1):61-7.

[3]. Cocker M, Francis R, Narici M, Birrell F. Sarcopaenia is highly prevalent in the very elderly and predicts mortality in males. Rheumatology. 2010;49:i62-i3.

[4]. Di Monaco M, Vallero F, Di Monaco R, Tappero R. Prevalence of sarcopenia and its association with osteoporosis in 313 older women following a hip fracture. Archives of gerontology and geriatrics. 2011;52(1):71-4.

[5]. Albala C, Lera L, Sanchez H, Angel B, Fuentes A, Arroyo P. Sarcopenia is more important than vitamin D deficiency as determinant of osteoporosis in chilean elders. Osteoporosis International. 2012;23:S343-S4.

[6]. Falutz J, Rosenthall L, Guaraldi G. Association of osteoporosis and sarcopenia in treated HIV patients. Antiviral Therapy. 2013;18:A17.

[7]. Go SW, Cha YH, Lee JA, Park HS. Association between Sarcopenia, Bone Density, and Health-Related Quality of Life in Korean Men. Korean journal of family medicine. 2013;34(4):281-8.

[8]. Miyakoshi N, Hongo M, Mizutani Y, Shimada Y. Prevalence of sarcopenia in Japanese women with osteopenia and osteoporosis. Journal of bone and mineral metabolism. 2013;31(5):556-61.

[9]. Scott D, Aitken D, Ebeling PR, Sanders K, Hayes A, Jones G. 'Sarco-osteoporosis': The prevalence and functional outcomes of comorbid sarcopenia and osteoporosis in community-dwelling older adults. Osteoporosis International. 2013;24:S563.

[10]. Sjoblom S, Suuronen J, Rikkonen T, Honkanen R, Kroger H, Sirola J. Relationship between postmenopausal osteoporosis and the components of clinical sarcopenia. Maturitas. 2013;75(2):175-80.

[11]. Verschueren S, Gielen E, O'neill TW, et al. Sarcopenia and its relationship with bone mineral density in middle-aged and elderly European men. Osteoporosis international : a journal established as result of cooperation between the European Foundation for Osteoporosis and the National Osteoporosis Foundation of the USA. 2013;24(1):87-98.

[12]. Huh JH, Song MK, Park KH, et al. Gender-specific pleiotropic bone-muscle relationship in the elderly from a nationwide survey (KNHANES IV). Osteoporosis International. 2014;25(3):1053-61.

[13]. Kim JE, Lee YH, Huh JH, Kang DR, Rhee Y, Lim SK. Early-stage chronic kidney disease, insulin resistance, and osteoporosis as risk factors of sarcopenia in aged population: the fourth Korea National Health and Nutrition Examination Survey (KNHANES IV), 2008-2009. Osteoporosis international : a journal established as result of cooperation between the European Foundation for Osteoporosis and the National Osteoporosis Foundation of the USA. 2014;25(9):2189-98.

[14]. Kim S, Won CW, Kim BS, Choi HR, Moon MY. The association between the low muscle mass and osteoporosis in elderly Korean people. Journal of Korean medical science. 2014;29(7):995-1000.

[15]. Albala C, Lera L, Sanchez H, et al. Osteoporosis, sarcopenia and fractures in chilean older people. Osteoporosis International. 2015;26(1):S332.

[16]. Wang YJ, Wang Y, Zhan JK, et al. Sarco-osteoporosis: Prevalence and association with frailty in Chinese community-dwelling older adults. International Journal of Endocrinology. 2015;2015.

[17]. Chung SM, Hyun MH, Lee E, Seo HS. Novel effects of sarcopenic osteoarthritis on metabolic syndrome, insulin resistance, osteoporosis, and bone fracture: the national survey. Osteoporosis international : a journal established as result of cooperation between the European Foundation for Osteoporosis and the National Osteoporosis Foundation of the USA. 2016;27(8):2447-57.

[18]. El Maghraoui A, Ebo'o FB, Sadni S, Majjad A, Hamza T, Mounach A. Is there a relation between pre-sarcopenia, sarcopenia, cachexia and osteoporosis in patients with ankylosing spondylitis? BMC musculoskeletal disorders. 2016;17:268.

[19]. Hars M, Biver E, Chevalley T, et al. Low Lean Mass Predicts Incident Fractures Independently From FRAX: a Prospective Cohort Study of Rece nt Retirees. Journal of bone and mineral research : the official journal of the American Society for Bone and Mineral Research. 2016;31(11):2048-56.

[20]. He H, Liu Y, Tian Q, Papasian CJ, Hu T, Deng HW. Relationship of sarcopenia and body composition with osteoporosis. Osteoporosis international : a journal established as result of cooperation between the European Foundation for Osteoporosis and the National Osteoporosis Foundation of the USA. 2016;27(2):473-82.

[21]. Hong S, Choi WH. The effects of sarcopenia and obesity on femur neck bone mineral density in elderly Korean men and women. Osteoporosis and sarcopenia. 2016;2(2):103-9.

[22]. Lee DW, Choi EY. Sarcopenia as an Independent Risk Factor for Decreased BMD in COPD Patients: Korean National Health and Nutrition Examination Surveys IV and V (2008-2011). PloS one. 2016;11(10):e0164303.

[23]. Yoshimura N, Muraki S, Oka H, et al. Is osteoporosis a predictor for future sarcopenia or vice versa? Four-year observations between the second and third ROAD study surveys. Osteoporosis international : a journal established as result of cooperation between the European Foundation for Osteoporosis and the National Osteoporosis Foundation of the USA. 2016;28(1):189-99.

[24]. Di Monaco M, Castiglioni C, Di Monaco R, Tappero R. Association between low lean mass and low bone mineral density in 653 women with hip fracture: does the definition of low lean mass matter? Aging clinical and experimental research. 2017;29(6):1271-6.

[25]. Frisoli A, Martim F, Borges J, Carvalho A, Chaves P. Association of Osteosarcopenia, Sarcopenia EWGSOP alone and osteoporosis alone with mobility in older adults: Data from sarcos study. Journal of the American Geriatrics Society. 2017;65:S49.

[26]. Frisoli A, Martin F, Ingham S, Carvalho AC. Body composition phenotype of osteosarcopenia, osteoporosis and sarcopenia: SARCOS study. Journal of Bone and Mineral Research. 2017;31.

[27]. Kim KM, Lee EY, Lim S, Jang HC, Kim CO. Favorable effects of skeletal muscle on bone are distinguished according to gender and skeletal sites. Osteoporosis and sarcopenia. 2017;3(1):32-6.

[28]. Krajewska-Wlodarczyk M, Owczarczyk-Saczonek A, Placek W. Changes in body composition and bone mineral density in postmenopausal women with psoriatic arthritis. Reumatologia. 2017;55(5):215-21.

[29]. Lee DW, Jin HJ, Shin KC, Chung JH, Lee HW, Lee KH. Presence of sarcopenia in asthma-COPD overlap syndrome may be a risk factor for decreased bone-mineral density, unlike asthma: Korean National Health and Nutrition Examination Survey (KNHANES) IV and V (2008-2011). International journal of chronic obstructive pulmonary disease. 2017;12:2355-62.

[30]. Locquet M, Beaudart C, Reginster JY, et al. Prevalence of Concomitant Bone and Muscle Wasting in Elderly Women from the SarcoPhAge Cohort: Preliminary Results. The Journal of frailty & aging. 2017;6(1):18-23.

[31]. Di Monaco M, Castiglioni C, Milano E, Massazza G. Is there a definition of low lean mass that captures the associated low bone mineral density? A cross-sectional study of 80 men with hip fracture. Aging clinical and experimental research. 2018;30(12):1429-35.

[32]. Frisoli A, Jr., Martin FG, Carvalho ACC, Borges J, Paes AT, Ingham SJM. Sex effects on the association between sarcopenia EWGSOP and osteoporosis in outpatient older adults: data from the SARCOS study. Archives of endocrinology and metabolism. 2018;62(6):615-22.

[33]. Hayashi F, Kaibori M, Sakaguchi T, et al. Loss of skeletal muscle mass in patients with chronic liver disease is related to decrease in bone mineral density and exercise tolerance. Hepatology research : the official journal of the Japan Society of Hepatology. 2018;48(5):345-54.

[34]. Hayashi M, Abe K, Fujita M, Okai K, Takahashi A, Ohira H. Association between sarcopenia and osteoporosis in chronic liver disease. Hepatology research : the official journal of the Japan Society of Hepatology. 2018;48(11):893-904.

[35]. Yoo JE, Park HS. Prevalence and associated risk factors for osteoporosis in Korean men. Archives of osteoporosis. 2018;13(1):88.

[36]. Du Y, Wang X, Xie H, et al. Sex differences in the prevalence and adverse outcomes of sarcopenia and sarcopenic obesity in community dwelling elderly in East China using the AWGS criteria. BMC endocrine disorders. 2019;19(1):109.

[37]. Kobayashi K, Ando K, Tsushima M, et al. Predictors of presarcopenia in community-dwelling older adults: A 5-year longitudinal study. Modern rheumatology. 2019;29(6):1053-8.

[38]. Lima RM, De Oliveira RJ, Raposo R, Neri SGR, Gadelha AB. Stages of sarcopenia, bone mineral density, and the prevalence of osteoporosis in older women. Archives of osteoporosis. 2019;14(1):38.

[39]. Locquet M, Beaudart C, Reginster JY, Bruyère O. Association Between the Decline in Muscle Health and the Decline in Bone Health in Older Individuals from the SarcoPhAge Cohort. Calcified tissue international. 2019;104(3):273-84.

[40]. Papageorgiou M, Sathyapalan T, Schutte R. Muscle mass measures and incident osteoporosis in a large cohort of postmenopausal women. Journal of cachexia, sarcopenia and muscle. 2019;10(1):131-9.

[41]. Reiss J, Iglseder B, Alzner R, et al. Sarcopenia and osteoporosis are interrelated in geriatric inpatients. Zeitschrift fur Gerontologie und Geriatrie. 2019;52(7):688-93.

[42]. Saeki C, Takano K, Oikawa T, et al. Comparative assessment of sarcopenia using the JSH, AWGS, and EWGSOP2 criteria and the relationship between sarcopenia, osteoporosis, and osteosarcopenia in patients with liver cirrhosis. BMC musculoskeletal disorders. 2019;20(1):615.

[43]. Taniguchi Y, Makizako H, Kiyama R, et al. The Association between Osteoporosis and Grip Strength and Skeletal Muscle Mass in Community-Dwelling Older Women. International journal of environmental research and public health. 2019;16(7).

[44]. Di Monaco M, Castiglioni C, Bardesono F, Milano E, Massazza G. Sarcopenia, osteoporosis and the burden of prevalent vertebral fractures: a cross-sectional study of 350 women with hip fracture. European journal of physical and rehabilitation medicine. 2020.

[45]. Kirk B, Phu S, Brennan-Olsen SL, Bani Hassan E, Duque G. Associations between osteoporosis, the severity of sarcopenia and fragility fractures in community-dwelling older adults. European Geriatric Medicine. 2020.

[46]. Lera L, Angel B, Márquez C, Saguez R, Albala C. Software for the Diagnosis of Sarcopenia in Community-Dwelling Older Adults: Design and Validation Study. JMIR medical informatics. 2020;8(4):e13657.

[47]. Nielsen BR, Andersen HE, Haddock B, Hovind P, Schwarz P, Suetta C. Prevalence of muscle dysfunction concomitant with osteoporosis in a home-dwelling Danish population aged 65–93 years - The Copenhagen Sarcopenia Study. Experimental Gerontology. 2020;138.

[48]. Petermann-Rocha F, Chen M, Gray SR, Ho FK, Pell JP, Celis-Morales C. Factors associated with sarcopenia: A cross-sectional analysis using UK Biobank. Maturitas. 2020;133:60-7.

[49]. Yu X, Hou L, Guo J, et al. Combined Effect of Osteoporosis and Poor Dynamic Balance on the Incidence of Sarcopenia in Elderly Chinese Community Suburban-Dwelling Individuals. Journal of Nutrition, Health and Aging. 2020;24(1):71-7.

[50]. Lee DY, Shin S. Association of Sarcopenia with Osteopenia and Osteoporosis in Community-Dwelling Older Korean Adults: A Cross-Sectional Study. Journal of clinical medicine. 2021;11(1).

[51]. Petermann-Rocha F, Ferguson LD, Gray SR, et al. Association of sarcopenia with incident osteoporosis: a prospective study of 168,682 UK biobank participants. Journal of cachexia, sarcopenia and muscle. 2021;12(5):1179-88.

[52]. Saeki C, Oikawa T, Kanai T, et al. Relationship between osteoporosis, sarcopenia, vertebral fracture, and osteosarcopenia in patients with primary biliary cholangitis. European journal of gastroenterology & hepatology. 2020.

[53]. Tan YJ, Lim SY, Yong VW, et al. Osteoporosis in Parkinson's Disease: Relevance of Distal Radius Dual-Energy X-Ray Absorptiometry (DXA) and Sarcopenia. Journal of clinical densitometry : the official journal of the International Society for Clinical Densitometry. 2020.

[54]. M DIM, Castiglioni C, Bardesono F, Freiburger M, Milano E, Massazza G. Is sarcopenia associated with osteoporosis? A cross-sectional study of 262 women with hip fracture. European journal of physical and rehabilitation medicine. 2022;58(4):638-45.

[55]. Pan Y, Xu J. Association between muscle mass, bone mineral density and osteoporosis in type 2 diabetes. Journal of Diabetes Investigation. 2022;13(2):351-8.

[56]. Xing E, Wan C. Prevalence of and factors associated with sarcopenia among elderly individuals with hypertension. The Journal of international medical research. 2022;50(7):3000605221110490.
