# Supplementary material for: A pooled analysis of the association between sarcopenia and osteoporosis
Source: Medicine (Baltimore). 2022 Nov 18;101(46):e31692. doi: 10.1097/MD.0000000000031692 (PMC9678526; doi:10.1097/MD.0000000000031692)
Supplement: Supplementary file 4 [file medi-101-e31692-s004.pdf]

S2 Table. Subgroup analysis of the interaction risk between sarcopenia and osteoporosis

Interaction	Factor	No. of groups	RR (95% CI)	Heterogeneity: I^2 (%)	P
SP increases OP	**Study design**				
	Cross-sectional study	42	3.10 (2.25, 4.26)	93.5	0.000
	Cohort study	3	1.92 (1.43, 2.58)	27.7	0.251
	Case-control study	3	4.35 (1.86, 10.16)	0.0	0.750
	NA	3	2.91 (1.36, 6.19)	74.0	0.021
	**Gender**				
	Male	15	3.80 (2.52, 5.74)	89.4	0.000
	Female	19	1.89 (1.54, 2.33)	53.6	0.003
	Female and Male	17	3.57 (2.71, 4.70)	51.2	0.008
	**Sarcopenia definitions**				
	1- Baumgartner's	11	2.40 (1.77, 3.24)	65.4	0.001
	2- EWGSOP	15	3.66 (2.02, 6.62)	95.1	0.000
	3- AWGS	11	3.33 (2.16, 5.12)	68.9	0.000
	4-Others	14	2.74 (1.96, 3.83)	74.3	0.000
	**Region**				
	1-Europe	17	2.50 (1.88, 3.32)	69.7	0.000
	2-America	8	3.02 (1.46, 6.24)	92.9	0.000
	3-Asia	24	3.00 (2.35, 3.84)	67.6	0.000
	4-Oceania	1	8.71 (2.87, 26.43)	-	-
	5-Africa	1	4.38 (1.13, 17.01)	-	-
OP increases SP	**Study design**				
	Cross-sectional study	13	2.87 (2.19, 3.77)	52.0	0.015
	Cohort study	2	1.97 (1.07, 3.63)	65.3	0.090
	Case-control study	1	3.51 (1.07, 11.46)	-	-
	NA	2	1.77 (0.52, 6.03)	52.6	0.146
	**Gender**				
	Male	3	2.77 (1.46, 5.25)	0.0	0.538
	Female	5	2.82 (1.46, 5.46)	91.1	0.000
	Male and female	10	2.54 (1.91, 3.38)	31.0	0.161
	**Sarcopenia definitions**				
	1- Baumgartner's	4	3.23 (1.14, 9.20)	48.8	0.119
	2- EWGSOP	5	4.03 (3.34, 4.87)	0.0	0.452
	3- AWGS	5	2.20 (1.44, 3.36)	38.9	0.162
	4-Others	4	2.29 (1.40, 3.75)	75.1	0.007
	**Region**				
	1-Europe	3	2.27 (0.48, 10.62)	69.5	0.038
	2-America	4	3.14 (2.40, 4.11)	0.0	0.897
	3-Asia	10	2.04 (1.49, 2.79)	42.9	0.072
	4-Oceania	1	2.89 (1.16, 7.21)	-	-
