# Supplementary material for: A pooled analysis of the association between sarcopenia and osteoporosis
Source: Medicine (Baltimore). 2022 Nov 18;101(46):e31692. doi: 10.1097/MD.0000000000031692 (PMC9678526; doi:10.1097/MD.0000000000031692)

Fig. S1. Sensitivity analysis of the estimated effects of sarcopenia on osteoporosis risk. The analysis was performed by recalculating the pooled results of the primary analysis after excluding one study per iteration.

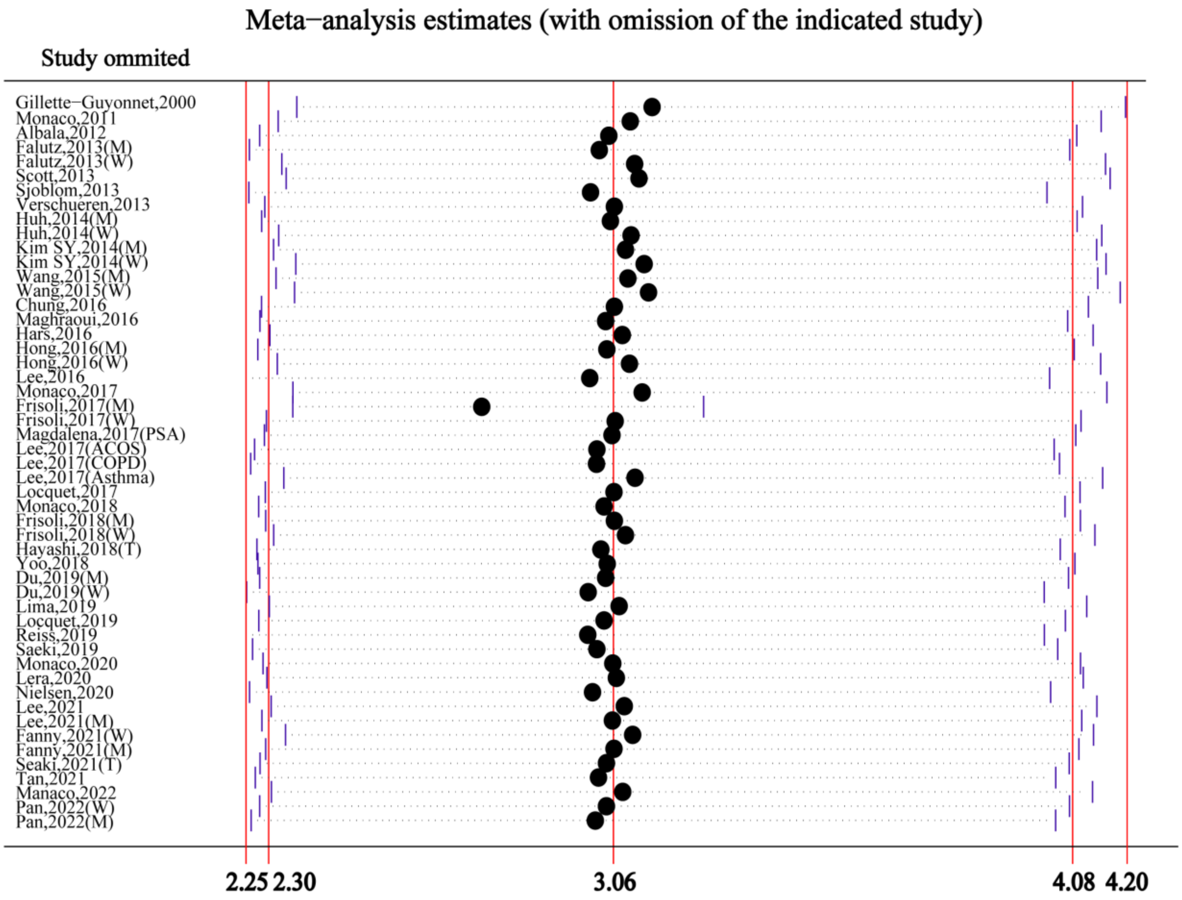

Fig. S2. Sensitivity analysis of the estimated decrease in osteoporosis risk associated with each SD increase in RASM. The analysis was performed by recalculating the pooled results of the primary analysis after excluding one study per iteration.

RASM: relative appendicular skeletal muscle mass

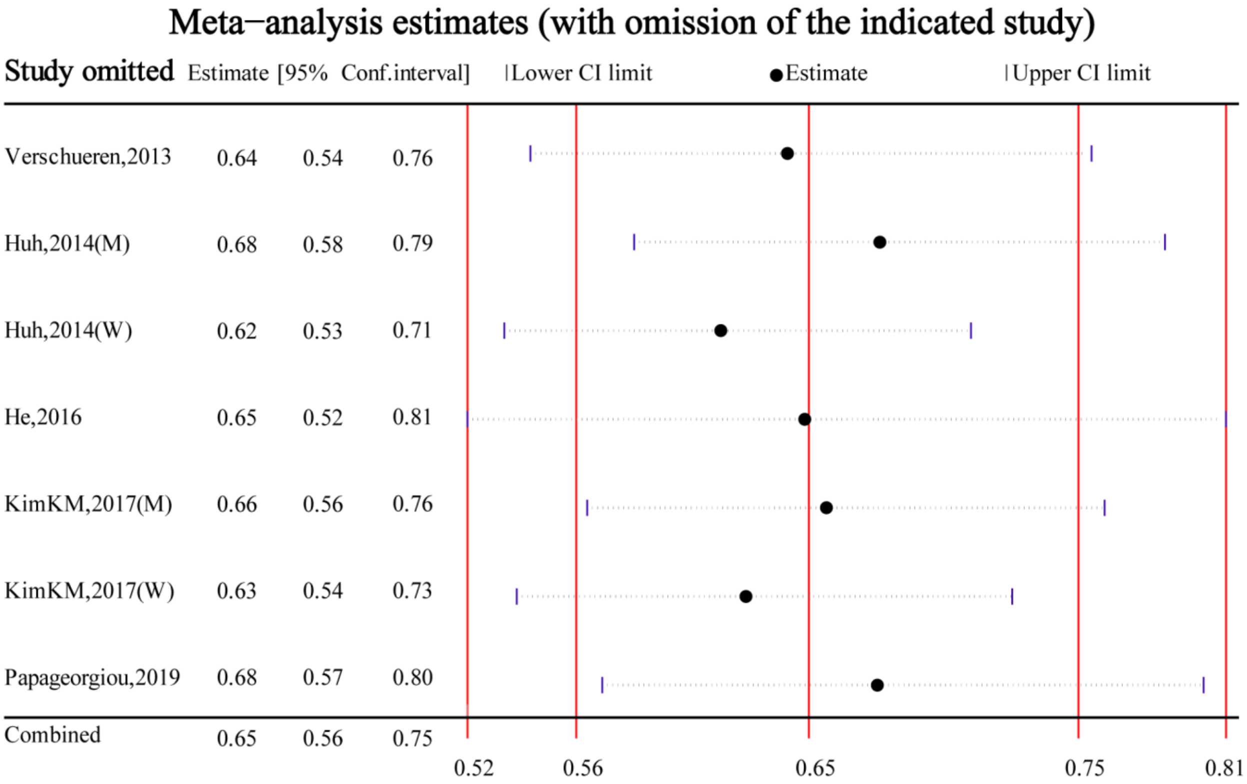

Supplement: Supplementary file 5 [file medi-101-e31692-s005.pdf]
