# Supplementary material for: A pooled analysis of the association between sarcopenia and osteoporosis
Source: Medicine (Baltimore). 2022 Nov 18;101(46):e31692. doi: 10.1097/MD.0000000000031692 (PMC9678526; doi:10.1097/MD.0000000000031692)
Supplement: Supplementary file 6 [file medi-101-e31692-s006.pdf]

Fig. S3. Publication bias among the studies that suggested that sarcopenia increases the risk of osteoporosis. A. Begg's rank correlation test was used to assess publication bias among the studies indicating that sarcopenia increases osteoporosis risk. B. The trim-and-fill method was used to assess publication bias among the studies that indicated that sarcopenia contributes to osteoporosis risk. C. Outcomes of the pooled analysis after the trim-and-fill method was used to assess publication bias among the studies that suggested that sarcopenia contributes to osteoporosis risk.

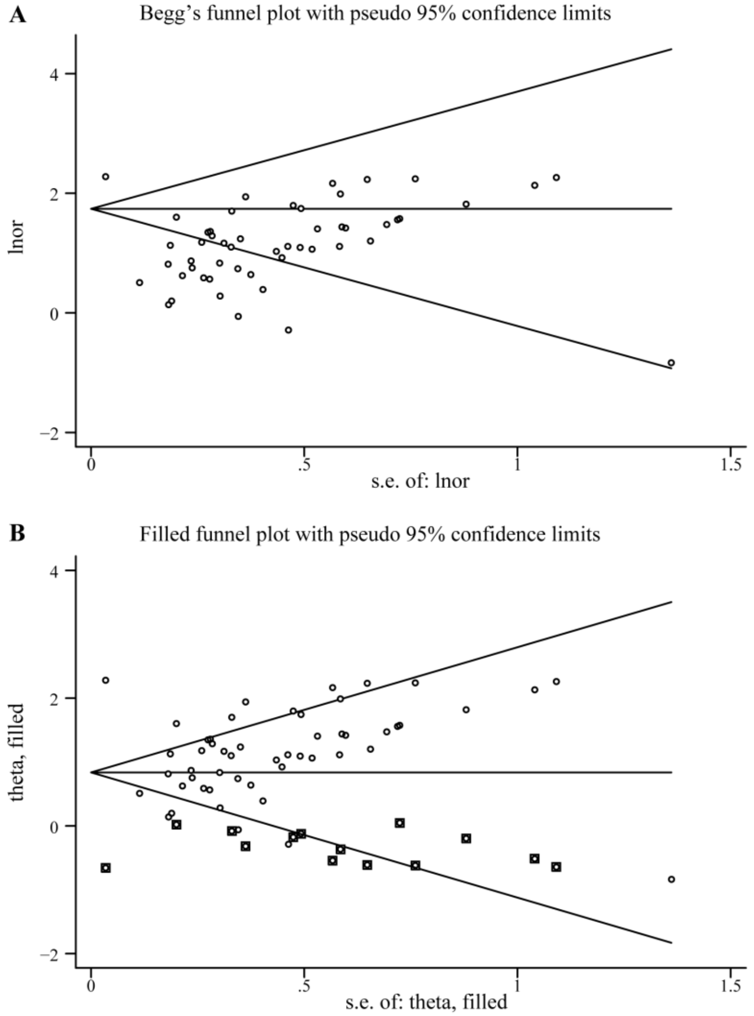

C

Method	Pooled Estimate	95% CI Lower	Upper	Asymptotic z_value	p_value	No. of Studies
Meta-analysis	1.740	1.687	1.793	64.583	0.000	51
Random	2.215	2.126	2.308	38.105	0.000	65

Filled
