# Supplementary material for: A pooled analysis of the association between sarcopenia and osteoporosis
Source: Medicine (Baltimore). 2022 Nov 18;101(46):e31692. doi: 10.1097/MD.0000000000031692 (PMC9678526; doi:10.1097/MD.0000000000031692)

Fig. S4. Sensitivity analysis of the estimated effects of osteoporosis on sarcopenia risk. The analysis was performed by recalculating the pooled results of the primary analysis after excluding one study per iteration.

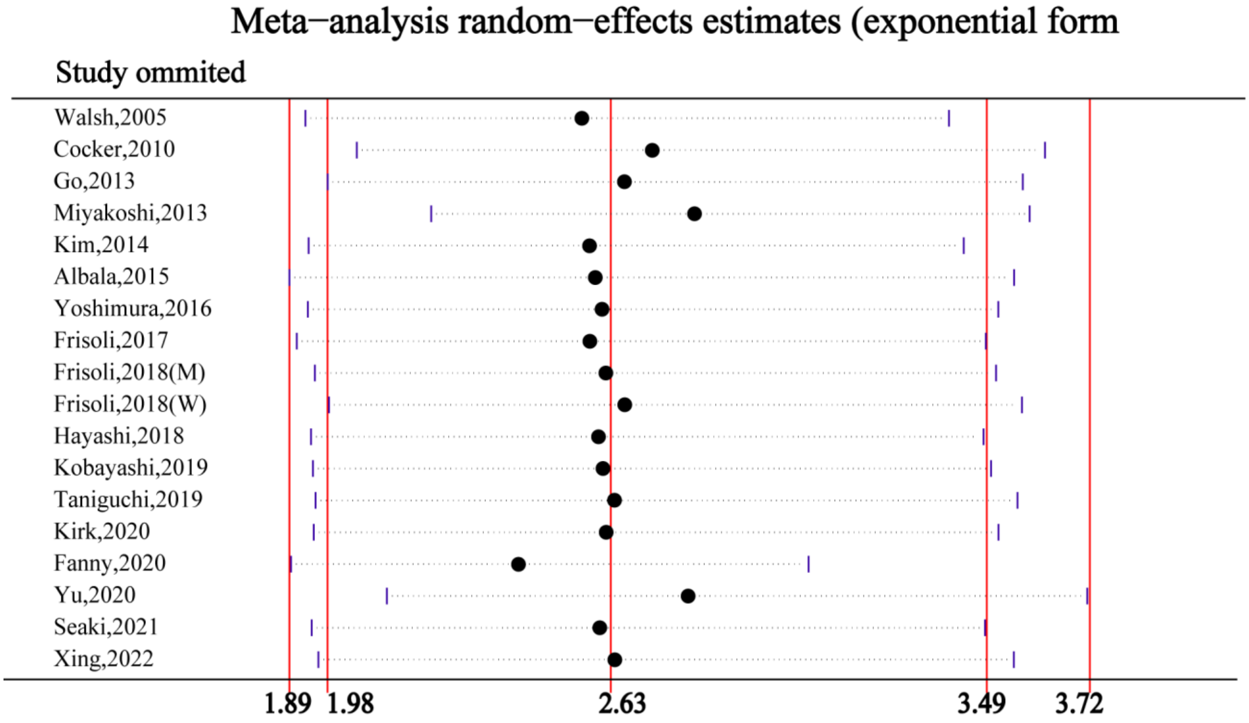

Supplement: Supplementary file 7 [file medi-101-e31692-s007.pdf]
