# Supplementary material for: A pooled analysis of the association between sarcopenia and osteoporosis
Source: Medicine (Baltimore). 2022 Nov 18;101(46):e31692. doi: 10.1097/MD.0000000000031692 (PMC9678526; doi:10.1097/MD.0000000000031692)

Fig. S5. Publication bias among the studies that indicated that osteoporosis increases sarcopenia risk. Begg's rank correlation test indicated a lack of publication bias among the studies that suggested that osteoporosis increases sarcopenia risk.

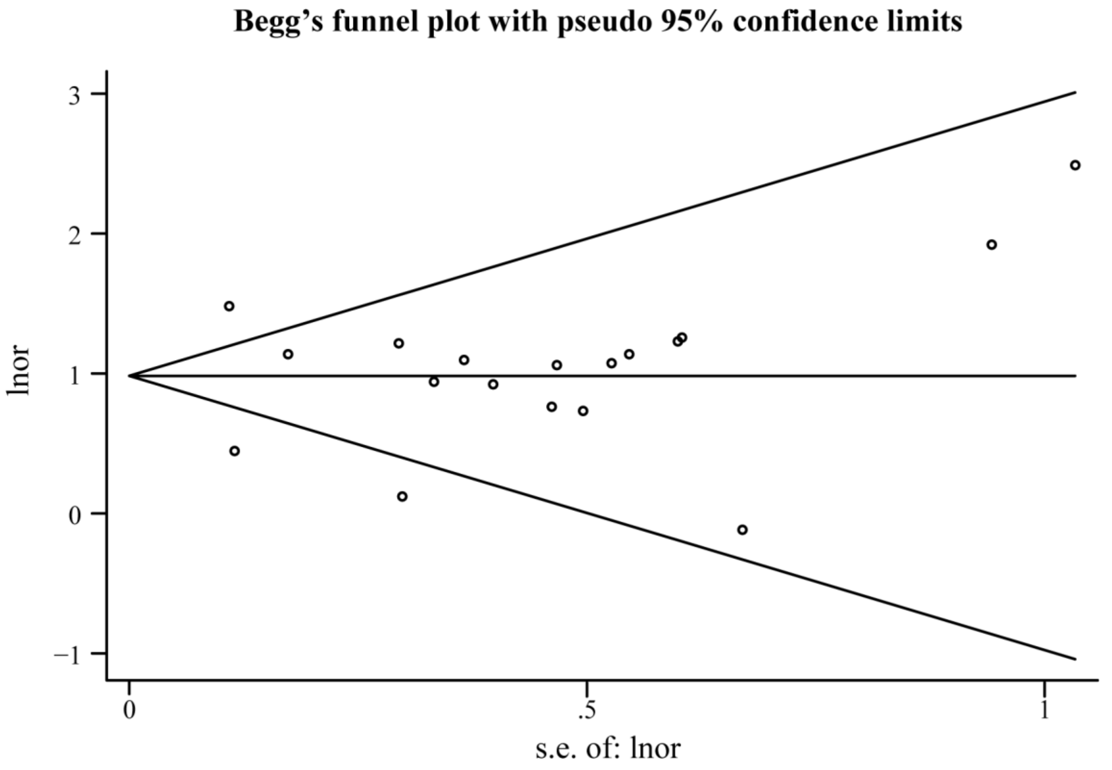

Supplement: Supplementary file 8 [file medi-101-e31692-s008.pdf]
